# Characteristics of Biopeptides Released In Silico from Collagens Using Quantitative Parameters

**DOI:** 10.3390/foods9070965

**Published:** 2020-07-21

**Authors:** Anna Iwaniak, Piotr Minkiewicz, Monika Pliszka, Damir Mogut, Małgorzata Darewicz

**Affiliations:** University of Warmia and Mazury in Olsztyn, Faculty of Food Science, Chair of Food Biochemistry, Pl. Cieszyński 1, 10-719 Olsztyn-Kortowo, Poland; minkiew@uwm.edu.pl (P.M.); monika.pliszka@uwm.edu.pl (M.P.); damir.mogut@uwm.edu.pl (D.M.); darewicz@uwm.edu.pl (M.D.)

**Keywords:** ADMET, BIOPEP-UWM database, bioactive peptides, collagen, in silico proteolysis, target prediction

## Abstract

The potential of collagens to release biopeptides was evaluated using the BIOPEP-UWM-implemented quantitative criteria including the frequency of the release of fragments with a given activity by selected enzyme(s) (A_E_), relative frequency of release of fragments with a given activity by selected enzyme(s) (W), and the theoretical degree of hydrolysis (DH_t_). Cow, pig, sheep, chicken, duck, horse, salmon, rainbow trout, goat, rabbit, and turkey collagens were theoretically hydrolyzed using: stem bromelain, ficin, papain, pepsin, trypsin, chymotrypsin, pepsin+trypsin, and pepsin+trypsin+chymotrypsin. Peptides released from the collagens having comparable A_E_ and W were estimated for their likelihood to be bioactive using PeptideRanker Score. The collagens tested were the best sources of angiotensin I-converting enzyme (ACE) and dipeptidyl peptidase IV (DPP-IV) inhibitors. A_E_ and W values revealed that pepsin and/or trypsin were effective producers of such peptides from the majority of the collagens examined. Then, the SwissTargetPrediction program was used to estimate the possible interactions of such peptides with enzymes and proteins, whereas ADMETlab was applied to evaluate their safety and drug-likeness properties. Target prediction revealed that the collagen-derived peptides might interact with several human proteins, especially proteinases, but with relatively low probability. In turn, their bioactivity may be limited by their short half-life in the body.

## 1. Introduction

Collagen is an extracellular protein being the structural component of connective tissues like skin, bone, cartilage, and tendons [1]. Its content is about 25–30% of the total human body protein [2]. According to the literature, there are at least 27 types of collagen [3], among which types I to V are the most common [4].

The structural nature of collagen was described by Gómez-Guillén et al. [5]. Briefly, it consists of three α-chains forming a triple helix stabilized by hydrogen bonds [5]. Moreover, all collagen types contain the G-P-Hyp repetitive sequential motif, where G stands for glycine, P for proline (mostly), and Hyp for hydroxyl-proline/hydroxyl-lysine. This motif is responsible for the triple helical structure and rigidity of the molecule [4].

According to Offengenden et al. [3], native collagen is somewhat resistant to the action of proteolytic enzymes. Collagen extracted with hot water from the source material is called gelatin [1]. Depending on acidic or alkaline conditions of the extraction, gelatin (i.e., degraded collagen) can be called type A or B gelatin, respectively [3]. The collagen-originating materials used for gelatin production include porcine skin, bovine hide, and bones [1]. Moreover, it was evidenced that collagen derived from porcine by-products shows high resemblance to the human collagen. Thus, there are no allergenic restrictions for using it, e.g., in skin and wound healing as well as plastic or reconstructive surgery. In turn, marine-derived collagens became the focus of particular interest due to their low inflammatory response, immunogenicity, as well as fewer ethical and religious barriers [6]. Also, great attention among scientists has been given to collagen/gelatin originating from ovine tendon and skin as well as chicken, duck, and rabbit skin [7].

It is generally well-known that peptides derived from different food proteins exhibit a variety of biological and physiological functions, including e.g., antihypertensive, antioxidative, immunomodulating, antibacterial [8], taste-affecting etc. [9]. The biological function of individual peptides is also related to the inhibition of: angiotensin I-converting enzyme (ACE; EC 3.4.15.1), dipeptidyl peptidase IV (DPP-IV; EC 3.4.14.5), α-glucosidase (EC 3.2.1.20), α-amylase (EC 3.2.1.1) [10], and lipase (EC 3.1.1.3) [11]. The ACE-inhibiting activity of peptides contributes to the blood pressure reduction in humans and animals [12], while inhibitors of lipase are involved in combating obesity [11]. In contrast, other aforementioned enzyme inhibitors are involved in the regulation of blood sugar level (antidiabetic peptides) [10]. Some of the biopeptides are used as components of nutraceutical foods due to their biological effect confirmed on humans. Considering the above, food proteins and their hydrolysates are in the focus of scientific interest as the health-beneficial food components useful in the prevention of diet-related diseases [13].

The use of enzymes (e.g., alcalase, pepsin, papain) for gelatin hydrolysis contributes to the production of collagen-originating peptides with molecular weights ranging from 3 to 6 kDa. This mixture of collagen-derived peptides is called a collagen hydrolysate (CH) [7]. According to the literature, peptides found in CH exhibited antioxidative and antimicrobial effects. Moreover, their function was to bind calcium, which in turn promoted their bioavailability. These properties of collagen-originating peptides allowed collagen to be considered as a valuable and functional food supplement [7].

One of the research trends concerning bioactive peptides relates to the involvement of in silico tools for their analysis. These in silico tools include, e.g., databases of protein and peptide sequences [14,15], programs for the prediction of the physicochemical properties of a peptide [16] and its bioactivity [17], and/or programs enabling the theoretical hydrolysis of protein aimed to produce peptides [18]. Another important field of study concerning bioactive peptides is related to the prediction of the bioactivity of a molecule (i.e., peptide) based on its structure. Such an approach is called QSAR, meaning the quantitative structure-activity relationship [19]. QSAR studies use the data that can be found in both bio- and cheminformatic databases as well as involve multivariate analyses [18]. According to Tu et al. [20], bioinformatics integrates many areas of “omics” sciences like proteomics, foodomics, transcriptomics, and metabolomics. Moreover, the application of bioinformatic-assisted methods allows minimizing the number of laboratory trials when analyzing the bioactivity of peptides based on their structure [20].

To recapitulate, the application of bio- and cheminformatics can be supportive when evaluating biopeptides and their protein sources. It can also prove helpful in understanding some phenomena when analyzing a massive amount of data. Taking into account the growing scientific interest in the analysis of biological functions of collagen hydrolysates as well as possibilities of studying biomolecules using bioinformatic tools, the aim of this study was the bioinformatic comparison of food protein-derived collagen sequences, including their “in silico” hydrolysates, as sources of biopeptides based on quantitative parameters.

## 2. Materials and Methods

Sequences of collagens (11), mainly collagen type I chains, were derived from the UniProt database of protein sequences (shortly, UniProt database) (providers: Swiss Institute of Bioinformatics, Lausanne, Switzerland and European Bioinformatics Institute, Hinxton, UK) [21] (http:/www.uniprot.org). They represented collagens derived from cow (*Bos taurus;* P02453), pig (*Sus scrofa;* A0A287BLD2), sheep (*Ovis aries;* W5P481), chicken (*Gallus gallus;* P02457), duck (*Anas platyrhynchos platyrhynchos;* A0A493T0N1), horse (*Equus caballus;* F6SSG3), salmon (*Salmo salar;* A0A1S3S6G4), rainbow trout (*Oncorhynchus mykiss;* O93484), goat (*Capra hircus;* A0A452FHU9), rabbit (*Oryctolagus cuniculus;* A0A5F9CPN0), and turkey (*Meleagris gallopavo;* G1NB83). Their UniProt accession numbers are provided in the brackets. All sequences of collagens (excluding signal peptide) were analyzed using the following procedure available in the BIOPEP-UWM database of protein and bioactive peptide sequences (shortly, BIOPEP-UWM database) (provider: University of Warmia and Mazury in Olsztyn, Poland) [22]: BIOPEP-UWM → Bioactive peptides or Proteins tab → Analysis → Calculations → For your sequence → *paste the collagen sequence* → Report. This procedure enabled the calculation of parameter A showing the potential of the protein to be the source of bioactive peptides.

In turn, the following procedure was applied to obtain the values of the quantitative parameters describing in silico proteolysis: BIOPEP-UWM → Bioactive peptides or Proteins tab → Analysis → Enzyme(s) action → For your sequence → *paste the collagen sequence* → Select enzymes (e.g., papain) → View the report with the results → tabs: Search for active fragments and Calculate A_E_, DH_t_, and W. The mathematical formulae of all numerical parameters mentioned above were introduced in detail by Minkiewicz et al. [22,23] and are provided in the Abbreviations section at the end of this article. Moreover, their descriptions can be found when opening: BIOPEP-UWM → any BIOPEP-UWM database tab → Analysis → Definitions → Calculations.

The following enzymes were used for the computer simulation of collagen hydrolysis using the procedure above: stem bromelain (EC 3.4.22.32), ficin (EC 3.4.22.3), papain (EC 3.4.22.2), pepsin (EC 3.4.23.1), trypsin (EC 3.4.21.4), and chymotrypsin (EC 3.4.21.1). The latter three were also applied in the following combinations: pepsin+trypsin and pepsin+trypsin+chymotrypsin, to show the simplified simulation of gastric and gastrointestinal digestion of the collagens, respectively.

PeptideRanker (provider: University College Dublin, Ireland) available at http://distilldeep.ucd.ie/PeptideRanker/ was applied to compute the likelihood of the released peptides to be bioactive (PeptideRanker Score) [17]. All in silico analyses were carried out in March–May 2020.

Putative interactions of the selected ACE- as well as DPP-IV-inhibiting peptides with human enzymes and other proteins were predicted using the SwissTargetPrediction web-tool (provider: Swiss Institute of Bioinformatics, Lausanne, Switzerland) [24], available at http://www.swisstargetprediction.ch/. The Simplified Molecular Input Line Entry Specification (SMILES) strings [25] of peptides, used as the input for the program, were constructed and verified according to the recommendations published in our previous article [26]. Amino acid sequences of peptides were converted into SMILES strings using “SMILES” application at the BIOPEP-UWM website [22]. Negative electric charges of acidic groups and positive charges of basic groups, characteristic of neutral pH, were introduced using a molecule editor Marvin JS (ChemAxon, Budapest, Hungary), available at the SwissTargetPrediction website. SMILES strings of nine peptides subjected to the cheminformatic analysis are presented in Table S1 of the Supplement.

The following additional properties were calculated for peptides: fulfilling Rule of 5 according to Lipinski et al. [27], Caco-2 permeability according to Wang et al. [28], human intestinal absorption according to Wang et al. [29], volume distribution according to Kerns and Di [30], half-life time according to Kerns and Di [30], as well as LD_50_ (lethal dose for 50% animals tested) of acute toxicity according to Lei et al. [31]. Calculations were performed using the ADMETlab platform (provider: Central South University, YueLu District, China) [32] available at the website: http://admet.scbdd.com/. SMILES representations, including ionization, were used as the input.

**Figure 1:**
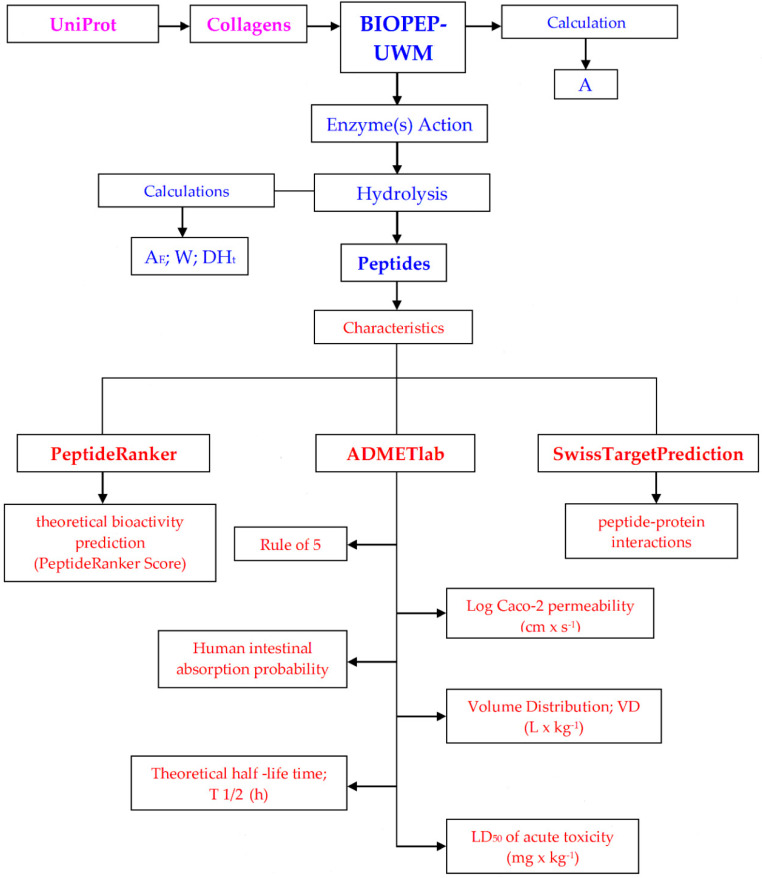
Workflow presenting the steps required to characterize collagen-derived peptides.

All steps required to characterize collagen-derived peptides are summarized in Figure 1.

Abbreviations: pink—steps involving the application of UniProt; blue—steps involving the application of BIOPEP-UWM database tools; red—steps involving the application of other tools to characterize biopeptides. 

## 3. Results and Discussion

The values of the frequency of the occurrence of peptides with a given activity (parameter A) in the collagens are shown in Table 1. The A values presented in this table were divided into equal and/or higher than 0.500 and described as “major A”. Other A values, i.e., those ranging from 0.100 to 0.499 and from 0.000 to 0.099, were called “moderate” and “minor” A, respectively. The superscripts assigned to each A value represent the specific code describing the bioactivity of a peptide in the BIOPEP-UWM database [22]. For example, A = 0.834 ^ah^ means that the frequency of the occurrence (A) of peptides with ACE-inhibiting effect (^ah^) was 0.834.

The parameter A is the quantitative criterion of protein evaluation that answers the following question: which bioactivities of peptides are encrypted in a protein sequence? It allows finding out relatively quickly and easy which bioactive fragments occur in the protein but does not indicate the particular sequence motifs as well as their location in the protein chain, which is the qualitative criterion of protein assessment called the profile of potential biological activity of a protein [22]. Thus, the A parameter enables a quick comparison of proteins’ potential as the source of bioactive components (i.e., peptides) according to the following rule: the higher the A value is, the better source of peptides with a given activity the protein is. The usefulness of parameter A was confirmed by Panjaitan et al. [33], who applied the proteomic approach to study the potential of giant grouper (*Epinephelus lanceolatus*) roe proteins as sources of peptides with ACE/DPP-IV inhibitory and antioxidant properties.

The values of parameter A were divided into three categories, namely: major, moderate, and minor A. For example, A ≥ 0.500 assumes that peptides exhibiting particular activity match minimum half of a protein chain understood as the total number of amino acid residues forming peptides compared to the total length of the protein chain. Such a way of understanding enables another assumption, namely that the major A suggests the high probability for the enzymatic release of peptides with such an activity from the protein of interest. A similar solution concerning the categorization of parameters taking into account their values was applied by Mooney at al. [17], who developed a tool called PeptideRanker, which serves to estimate peptides’ bioactivity. In the present study, the bioactivity of peptides was estimated using a theoretical parameter called PeptideRanker Score, whose values range from 0 to 1. According to the interpretation of the PeptideRanker Score, the higher its value is, the more likely the peptide tends to be bioactive. Moreover, PeptideRanker Score >0.5 indicates peptide’s potential to exhibit any bioactivity [17].

The predominant activities of all collagen sequences analyzed based on A values were related to ACE- and DPP-IV inhibition (see Table 1). The A value determined for the ACE inhibitory activity ranged from 0.799 (protein source: salmon) to 0.847 (protein source: pig, duck). In the case of collagens’ potential as the sources of DPP-IV inhibitors, A value ranged from 0.798 (protein source: salmon) to 0.870 (protein source: duck). Collagen derived from the horse had identical A values computed for both these bioactivities (A = 0.843).

ACE inhibitors are involved in blood pressure reduction, and many of them were identified in different food sources [12]. Peptides with the ACE-inhibiting effect are also the most extensively studied group of sequences considering their mechanism of action, structural character, identification of proteins, and the blood pressure-reducing effect analyzed both in humans and animals [12]. The structural characterization of these peptides involved the analysis of the impact of amino acid composition on their ACE-inhibiting activity. According to the literature, ACE inhibitors are usually composed of Gly, Ile, Leu, Val (N-terminus) and Pro, Tyr, Trp (C-terminus) [34,35]. Studies on the structure-function relationships of DPP-IV inhibitors (known as the regulators of blood sugar level and, thus, antidiabetic peptides) have shown that the presence of Trp at N-terminus and of Pro at the second position of a peptide sequence was correlated with relatively good potency of these peptides. Moreover the opposite sequential order of these amino acids yielded a relatively high DPP-IV inhibitory effect expressed by their low IC_50_ values (i.e., concentration of a peptide corresponding to its half-inhibitory effect) [36]. This specific amino acid composition of peptidic ACE- and/or DPP-IV inhibitors affected their match to their collagen precursors. It is well-known that Gly and Pro are the major amino acids in collagen sequences [37].

Moderate A values indicated that all collagens were potential sources of peptides with antiamnestic, antithrombotic, and regulating properties, the latter of which included the regulation of: cell permeability, ion flow, mechanism of phosphoinositol action heart muscle contraction, as well as the activation of stomach mucosa membrane and/or phosphatase and kinase. The occurrence of peptides with one of the aforementioned activities was rare. Thus they were summarized as “regulatory” peptides. Generally, the value of parameter A for these activities did not exceed 0.300. The highest values of moderate A were obtained for collagens from goat (A = 0.273 for antithrombotic activity) and pig (A = 0.216 both for antiamnestic and regulating activities). The lowest values of moderate A were observed for collagen derived from rainbow trout (A = 0.161 and 0.162 for antiamnestic and regulating activity, respectively) and salmon (A = 0.170 for antithrombotic activity).

The lowest A values (minor A) described the weak potential of collagens as the sources of peptides capable of inhibiting dipeptidyl peptidase III (DPP-III; EC 3.4.14.4), α-glucosidase, renin (EC 3.4.23.15), and other enzymes. Other activities included, e.g., immunomodulating, activating ubiquitin-mediated proteolysis, antioxidative, bacterial permease ligand, and hypolipidemic effect (for details, see Table 1). The A values ranged between 0.001 (embryotoxic, bacterial permease ligand, immunomodulating activities) and 0.083 (DPP-III inhibitor).

The potential of collagens as the sources of biopeptides was studied using in silico and in vitro approaches [38]. The first includes the analysis using databases for peptide screening and engages computer tools to predict collagens’ potential to hydrolyze proteins with enzymes to produce biopeptides. The second is a combination of theoretical predictions (in silico approach) and in vitro experiment involving the hydrolysis of collagens and then the characteristics of released peptides using mass spectrometry [38,39].

In silico analyses are becoming more popular among scientists who work on bioactive peptides from foods [40]. One of the research trends involves the computer simulation of protein hydrolysis [41]. The BIOPEP-UWM database, which offers a tool for predicting peptides that may be released from protein, has so far been used to predict specific sequences. Such a prediction enabled defining “known peptides” in the “new proteins” or extending the knowledge on proteins as sources of “new peptides” (i.e., not identified so far in the protein of interest). Regardless of the type of prediction, attempts were made to identify the sequences in protein hydrolysates experimentally. Such an approach was applied by, e.g., Borawska et al. [42] to identify antioxidative and ACE inhibitory peptides in ex vivo hydrolysates of carp (*Cyprinus carpio*) muscle tissue.

As mentioned above, bioactive peptides are produced, e.g., via the enzymatic hydrolysis of proteins [43]. Different enzymes are involved in producing peptides from proteins, including collagens, namely: bromelain, ficin, papain, pepsin, trypsin, and chymotrypsin [38]. These enzymes were used in our study to analyze the theoretical potential of collagens to produce bioactive peptides as well as to observe the potential of the proteases when generating peptides. It was possible due to the application of the three following quantitative parameters: A_E_, W, and DH_t_. Descriptors like A_E_ and W were introduced by Minkiewicz et al. [23], who used them to analyze the potential of bovine meat proteins as the sources of peptides. Currently, these parameters, along with some others (not applied in the present study), have been available in the BIOPEP-UWM database since 2019.

The results of the quantification of collagens using the aforementioned parameters are shown in Table 2. Parameter A_E_ (the frequency of the release of fragments with a given activity by the selected enzyme) suggests that a given enzyme can release bioactive fragments. The higher the A_E_ value, the higher the number of peptides with specific activity produced by the enzyme.

Referring this rule to our results, the highest potential was represented by bromelain (B) being the most effective enzyme that produces peptides with dipeptidyl peptidase IV-inhibiting activity. Its A_E_ value ranged from 0.141 (collagen from turkey) to 0.158 (collagens from: cow, pig, sheep, rabbit). Value A_E_ = 0.158 was also achieved for papain producing dipeptidyl peptidase IV inhibitors from chicken collagen. The lowest values of A_E_ and W provided in Table 2 were rounded to thousandths and could reach 0.001. These A_E_ values were determined for all enzymes used to stimulate the hydrolysis of all collagen sequences producing peptides with different bioactivities. These activities also included those described by minor A (see Table 1).

Another studied parameter, i.e., W, defines the relative frequency of release of fragments with a given activity by selected enzymes [22,23] and is complementary to the A_E_. Its high value suggests that a given enzyme contributes to the release of a high percentage of fragments with a given activity from the protein (i.e., collagen). Thus, the highest value of W was observed for peptidic bacterial permease ligands released using papain (source: bovine collagen) and for peptidic immunomodulators released using bromelain (source: ovine collagen). The immunomodulating bioactivity of the peptides potentially released from ovine collagen using bromelain was not revealed when calculating A_E_. This was also noticed in some other cases (for details, see Table 2), probably due to the A_E_ value being less than 0.001 and thus not included in Table 2.

Values of parameter A_E_ also showed that some enzymes are potentially able to release the same number of peptides from all collagens. It concerned mostly bromelain and ficin, and also pepsin+trypsin+chymotrypsin, all of them theoretically released peptides with antiamnestic, antithrombotic, and regulating activities. In the case of collagens derived from goat, horse, and rabbit, trypsin (A_E_ = 0.001) had an equal potential to produce antioxidative, antithrombotic, ACE- and DPP-IV inhibitory peptides (see Table 2). Activities of those of peptides that could be released from these collagens refer to oxidative stress regulation and their cardioprotective and antidiabetic potentials [44]. The values of A_E_ for these activities were 0.01 (caprine collagen) and 0.001 (equine and rabbit collagen). Peptides with similar potentials, i.e., antioxidative, α-glucosidase, and renin inhibitors (the latter two representing antidiabetic and antihypertensive effect, respectively), were also predicted to be released from rainbow trout (enzyme used: bromelain) at the equal potency (A_E_ = 0.003). Diabetes, hypertension, cardiovascular diseases, oxidative stress, obesity, and inflammation are the body dysfunctions related to the human diet and lifestyle. The co-occurrence of at least of three of these dysfunctions is called metabolic syndrome [44]. Thus, the prediction of the potency of some collagens to produce peptides that may affect the regulation of symptoms related to metabolic syndrome may help study proteins as the sources of multi-active peptides.

The theoretical degree of hydrolysis (DH_t_) shows the efficiency of the enzyme to produce peptides from collagens. The highest DH_t_ values were observed for the plant-derived enzymes, which is due to their broad specificity. The most efficient was the hydrolysis of rainbow trout collagen with bromelain (DH_t_ = 61.89%). The lowest DH_t_ values were typical of the animal-derived enzymes with a narrow specificity (e.g., DH_t_ = 4.03% of pepsin used for salmon collagen hydrolysis). Some of the aforementioned proteases were used in two- (pepsin+trypsin) or three-enzyme (pepsin+trypsin+chymotrypsin) combinations, which caused the increase of both DH_t_ value and the efficiency of the theoretical release of biopeptides. It is worth mentioning that the computer simulation of proteolysis assumes that all peptide bonds are hydrolyzed in a protein chain. This issue is more complicated when hydrolyzing the protein in laboratory conditions, as described by Iwaniak et al. [45,46] who hydrolyzed milk and soybean proteins as sources of bitter-tasting motifs using in silico and in vitro protocols.

When comparing both descriptors, their values can be explained as follows: the high value of A_E_ (being the result of high A value) and the low value of W may suggest that although the protein is a good (i.e., rich) source of biopeptides, the applied enzyme is rather useless in releasing peptides having specified functions from the specific protein. However, this rule did not apply to the collagen sequences analyzed. Usually, they were the “comparable A_E_ and W” or “lower A_E_ and higher W” variants. The first variant suggested that the protein might be a rich/poor source of some peptides and that the enzyme had the adequate potential to release them. The second variant meant that the enzyme applied could be efficient to produce peptides, however, the protein was instead a poor source of such peptides.

After analyzing both A_E_ and W values determined for the same activities of peptides theoretically generated from collagens, the next step was to establish the composition of the sequences released. Therefore, the variant “comparable A_E_ and W values” (see above), that was achieved for some collagens was selected for further analyses. Such values were achieved for 7 collagens (from: cow, pig, sheep, chicken, horse, salmon, trout) that were hydrolyzed using pepsin and/or trypsin to produce peptides being ACE and/or dipeptidyl peptidase IV inhibitors. The amino acid sequences of these peptides are given in Table 3. Then, the PeptideRanker Score parameter was calculated for all ACE- and DPP-IV inhibitors. The PeptideRanker Score was described by Mooney et al. [17] as the parameter available in this program and showing the likelihood for the peptide to be bioactive but without specifying the exact bioactivity that may be related to a given sequence. Values of this parameter range from 0 to 1 (the higher the PeptideRanker Score, the higher the likelihood for a peptide to be bioactive) [17]. According to Mooney et al. [17], it is presumed that a peptide with PeptideRanker Score >0.5 will show bioactivity in experimental conditions. According to our results, one peptide (DR, derived from horse collagen hydrolyzed with pepsin) had PeptideRanker Score = 0.289. The majority of peptides potentially released from these collagens were dipeptides showing dual bioactivity. Only one tripeptide, PGL—acting as the ACE inhibitor, was released due to the action of pepsin on the collagens derived from cow, pig, chicken, horse, and salmon. Short motifs, like di- and tripeptides, more easily match the sequence of the parent protein [45,46]. Considering the peptide sequences provided in Table 3, most of them were composed of the following amino acids: Pro, Phe, Gly, Leu, and Arg. According to Song et al. [47], the first three residues were found as peptide constituents characteristic of DPP-IV inhibitors, whereas Arg (among others) can be found in the peptides acting as ACE inhibitors [48].

This strategy of research based on searching for biopeptides with known sequences in the protein that so far had not been known as their source is called a positive selection. Such an idea to study biopeptides from foods was applied by several authors [45,46,49,50,51]. Thus, the next step in our study was to acquire the information about the peptides theoretically found in peptic and/or tryptic hydrolysates of collagens. The only tripeptide (PGL) was an ACE inhibitor (IC_50_ = 13.93 μM) that was identified in the gelatin of an Alaskan Pollack skin [52]. This peptide was theoretically identified in the peptic hydrolysate of salmon collagen (PeptideRanker Score = 0.855) and hydrolysates of cow, chicken, pig, and horse (see Table 3).

Five in silico peptic collagen hydrolysates (source: cow, pig, chicken, sheep, horse) contained dual-active peptides (ACE/DPP-IV inhibitors), namely SF and TF. The SF peptide was identified in aqueous garlic extracts (ACE inhibitor; IC_50_ = 130.2 μM) [53] and synthesized to show the DPP-IV-inhibiting activity (level of inhibition = 13.5%) [54]. In turn, the TF peptide was produced by autolysis of wheat milling by-products. It was identified in one of the heat bran fractions exhibiting the ACE inhibitory effect (IC_50_ = 18.0 μM) [55]. The DPP-IV-inhibiting potential of the TF sequence was confirmed when analyzing the library of dipeptides (level of inhibition = 32.1%) [54]. Apart from the in silico collagen hydrolysates mentioned above, the TF peptide was also theoretically identified in salmon collagen hydrolyzed by pepsin.

Another sequence, QF, was identified in the peptic hydrolysates of bovine and chicken collagens (see Table 3). It was discovered as a DPP-IV inhibitor (level of inhibition = 28.6%) [54]. In turn, DF peptide was confirmed to act as the ACE inhibitor. It was originally identified in anchovy fish sauce (IC_50_ = 360 μM) [56]. In the present study, the DF sequence was identified only in the equine collagen theoretically hydrolyzed by pepsin. This collagen was also a source of DR peptide (enzyme applied: trypsin), which was first reported by Lan et al. [54] as a DPP-IV inhibitor (level of inhibition = 26.1%). Moreover, the aforementioned sequence had the lowest PeptideRanker Score (0.286). In turn, the GR peptide (PeptideRanker Score = 0.766), a product of in silico tryptic hydrolysis of the collagen derived from rainbow trout, was reported in the literature as the ACE inhibitor (IC_50_ = 162.2 μM) produced by the action of several muscle protein-originating dipeptidyl peptidases which remain active during the whole period of dry-cured meat processing [57].

The presence of the RL sequence (PeptideRanker Score = 0.626) was observed in all peptic hydrolysates of collagens. This sequence possessed dual bioactivity. It was identified as the ACE inhibitor (IC_50_ = 2439 μM) (origin: β-lactoglobulin) [58] and human DPP-IV inhibitor (level of DPP-IV inhibition = 20.2%; library of synthetic peptides) [54]. Dual bioactivity was also exhibited by the GF sequence theoretically released from cow and sheep collagens (enzyme used: pepsin). This peptide showed the ACE inhibitory (IC_50_ = 277.9 μM; source: aqueous garlic extracts) [53] and the DPP-IV inhibitory effect [59]. The later bioactivity was confirmed for GF derived from residual meat of salmon digested with Corolase PP (IC_50_ = 1547 μM) [59]. This peptide had the highest PeptideRanker Score (0.994) among all peptides reported in the in silico hydrolysates of collagens (see Table 3).

As could have been noticed, all peptides, except one (DR), predicted to be products of theoretical hydrolysis of some collagens, were highly bioactive (high PeptideRanker Score values) and also showed the effect in vitro. However, the relatively high PeptideRanker Score was not always the parameter indicating the high bioactivity of a peptide determined in the laboratory conditions (e.g., RL with PeptideRanker Score = 0.626 but IC_50_ = 2.439 μM). Similar results were obtained by Fu et al. [50], who applied in silico analysis to assess the potential of patatin (*Solanum tuberosum*; potato) to release bioactive peptides. They showed that, e.g., FP peptide that was identified in the patatin sequence had a high PeptideRanker Score (0.99), but exhibited a relatively low ACE inhibitory activity (IC_50_ = 1215.7 μM; source of the peptide—Manchego cheese). Another patatin-encrypted peptide—WG—was also highly likely to be bioactive (PeptideRanker Score = 0.99) but no information on this peptide was found in the literature when analyzing the data. Thus, according to Fu et al. [50], although it is rather impossible to estimate the potency of a peptide to be bioactive using the bioinformatic tools like, e.g., PeptideRanker, such an approach may prove useful in the structure-activity relationship analyses. In turn, peptides with relatively the highest PeptideRanker Scores may be synthesized to determine their in vitro bioactivity.

Pripp et al. [60] highlighted the role of peptide bioactivity determinations in the studies concerning their quantitative structure-activity relationships (QSAR). A specific activity (e.g., ACE inhibition) can be determined by researchers using different methodologies and units, which affects the precision when constructing the QSAR models. Another reason behind differences between the PeptideRanker Scores and experimental bioactivity of peptides may be the biological effect estimation method. This point of view, although concerns the peptide QSAR modeling, can also refer to the possible differences between the theoretical (e.g., PeptideRanker Score) and experimental bioactivity of a peptide (e.g., IC_50_ value). Therefore, some authors postulate unifying the concentration units when determining the inhibitory activity and establishing a standard procedure to construct an updated real-time database [61].

The issue concerning the “parameters based on the frequency of the occurrence of peptides vs. their activity” is more complex, as discussed by Minkiewicz et al. [23]. It may happen that the release of a higher number of peptides with weak bioactivity will result in the stronger hydrolysate than that containing one peptide with a strong effect [23]. In our study, rather, the majority of in silico collagen hydrolysates contained peptides that were weaker in terms of their bioactivity expressed using the IC_50_ parameter. The relatively strong peptide was PGL with its ACE-inhibiting effect (IC_50_ = 13.93 μM), which was theoretically identified in bovine and salmon collagen peptic hydrolysates. Moreover, it is noteworthy that successful in silico estimation of peptides’ release from the proteins depends on the regular update of the database with the new sequences and/or completing the information about the bioactivities of peptides and specificity of enzymes [23,62].

As presented above, our approach shows how to determine the theoretical potential of selected collagens to produce biopeptides using quantitative parameters. It starts from the analysis of the potential of proteins as sources of peptides based on parameter A. Then, other descriptors, like A_E_, W, and DH_t_, are calculated, and finally, the proteins with the comparable values of A_E_ and W are selected for further analysis, including PeptideRanker Score computation. This procedure is one of the efficient templates to characterize proteins using high-throughput technologies (HTs). Briefly, HTs deal with the automatic data analysis in a timely manner, paying attention to data pre- and post-processing to get the reliable interpretation and annotation of the dataset [63]. HTs were applied to predict the antihypertensive potential of fish proteins using an AHTPDB, BIOPEP-UWM, PeptideCutter tool. Among 18 fish species analyzed, collagens were theoretically the rich sources of antihypertensive peptides, however, the application of pepsin and trypsin revealed that not all predicted sequences were released [61]. Discrepancies between in silico and in vitro analyses were also observed when producing ACE inhibitors from bovine collagens using papain. In silico hydrolysis of collagen sequences led to more than 100 ACE inhibitors (mostly dipeptides) being obtained. Short-length peptides were not identified in the most potent peptide fraction of the collagen hydrolysate. The mismatch between theoretically and experimentally produced peptides could be explained the complex spatial structure of collagen hindering the enzyme access to cleavage sites of native proteins [64]. Other authors also included the post-translational modifications as well as amino acid composition of collagen-derived peptides. Collagen is rich in hydroxyproline which might be “not recognized” by programs serving for theoretical hydrolysis [65]. The additional factors which are “not considered” by programs for bioinformatic-assisted hydrolysis were discussed by Iwaniak et al. [45,46] who applied the integrated approach for milk and soybean proteolysis. These factors included among others the complete characteristics of enzyme (optimal pH, temperature, enzyme-to-substrate ratio), complexity of protein structure, location of the enzyme and substrate in different extra- and intracellular regions, and involvement of inhibitors [45,46]. On the other hand, even if the proteolysis is incomplete, predicted peptides may be detected as judged during experiments carried out on milk [45] and soybean [46] proteins. To recapitulate, bioinformatic platforms enable identification of biopeptides in hydrolysates in silico and lead to the next step of research, namely physiological analyses [61]. This approach can also be employed in our procedure. However, it should be noted that the in silico analyses make the exploration of collagen-derived peptides relatively easy, but the limitations should not be ignored [38]. 

**Figure 2:**
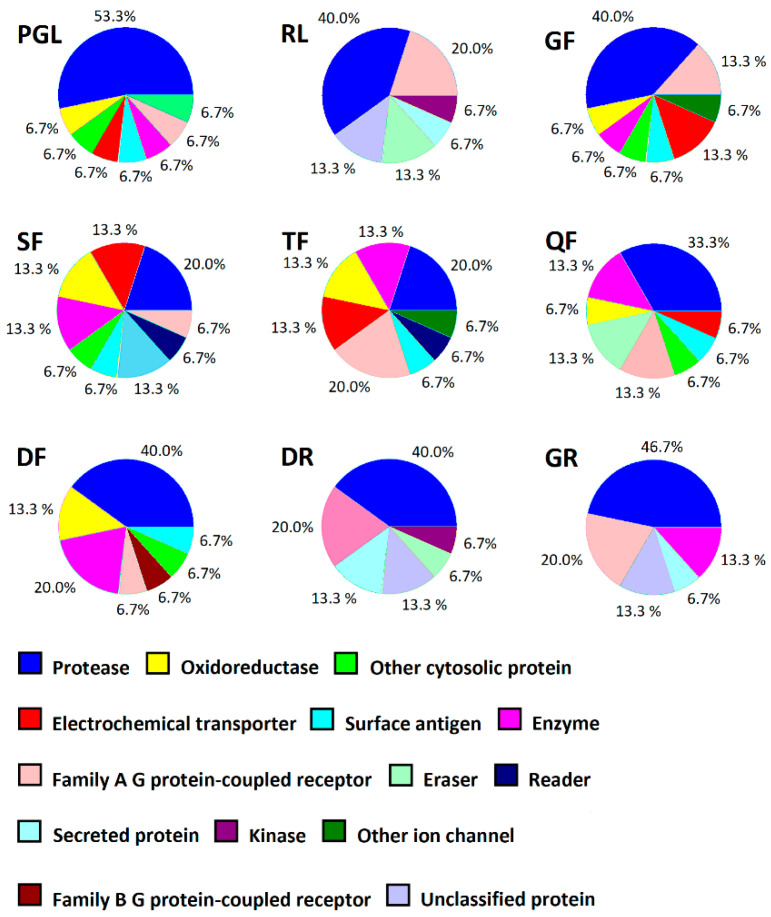
Classes of proteins (according to the classification provided by SwissTargetPrediction web-tool) potentially interacting with ACE and DPP-IV inhibitors theoretically released from collagens (15 most likely proteins, indicated in the Tables S2–S10 of the Supplement, in red fonts, were taken into account).

Many enzymes and other proteins are targets for the bioactive peptides [9], but usually, only a few peptides are known as the ligands of the individual protein. Hence, some computer programs offer useful tools to search for potential targets of a given short-length peptide. One such tool is SwissTargetPrediction [24], which enables the target predictions for compounds with low molecular mass (hundreds of Daltons), including di- and tripeptides. The program compares the structures and electric charge distribution of query compounds (in our case oligopeptides) and known protein ligands including enzyme inhibitors, small molecules which may be bound together with receptor proteins and ligands of transporters. SwissTargetPrediction web-tool utilizes a set of protein ligands (e.g., approved drugs) annotated in the ChEMBL database of molecules with drug-like properties (shortly, ChEMBL database) (provider: European Bioinformatics Institute, Hinxton, UK) [66]. The model used by program assumes that the higher structural similarity between molecules (including chirality and charge distribution) implies the higher probability that they reveal affinity to the same target protein (e.g., inhibit the same enzyme) [24]. The output shows the list of proteins potentially interacting with a given ligand (e.g., peptide), including the probability of interaction. In the case of food peptides SwissTargetPrediction was applied, e.g., to predict the interactions of anticancer peptides of plant origin [67,68]. Thus, in our study, all ACE and DPP-IV inhibitors that were potentially produced from collagens (see Table 3) were subjected to target prediction using this tool. The results of this analysis presenting the classes of proteins (15 most likely proteins) being the potential targets for particular ACE and DPP-IV inhibitors are summarized in Figure 2. The detailed information concerning the SwissTargetPrediction results is provided in Tables S2–S10 of the Supplement. SwissTargePrediction is designed as a tool supporting discovery and/or design of new drugs. The program provides also the classification of proteins being the potential targets of small molecules (see Figure 2). It should be noted that in the case of enzymes, the aforementioned classification does not reflect EC classification, recommended by the International Union of Biochemistry and Molecular Biology (IUBMB). Enzyme classes and subclasses, such as oxidoreductases (EC 1) proteinases (EC 3.4), or kinases (EC 2.7.10, 2.7.11, 2.7.12, 2.7.13, 2.7.14, and 2.7.99), being in the focus of the special attention of pharmaceutical sciences, are emphasized in program output (although they belong to different classification levels).

The most abundant classes of proteins potentially interacting with the aforementioned peptides were the enzymes, mainly the proteolytic ones. Other common protein classes were the receptors and transporters.

Table 4 annotates three proteins revealing the highest probabilities of interactions with a given peptide. Most of the probability values ranged from ca. 0.1 to ca. 0.24. The highest value of probability (0.526) was achieved for peptide PGL as a ligand of DPP-IV. To date, this peptide has not been known to exhibit DPP-IV inhibitory activity (see Table 3), but the result above suggests that it is a promising candidate in this respect. In turn, displaying ACE as a PGL sequence target agrees with its bioactivity determined experimentally. Our results revealed that PGL was also likely to be a ligand of cyclooxygenase-2 (COX-2; prostaglandin-endoperoxide synthase; EC 1.14.99.1). The proteins predicted to be among the three most likely ligands of at least two peptides are briefly described below.

The most abundant protein among the top three potential targets for peptides was the proteolytic enzyme calpain 1 (EC 3.4.22.52) (5 peptides in Table 4). Calpains (including calpain 1) are known as the modulators of cellular signaling. Their abnormal function is associated with neurodegenerative diseases, cancer, limb-girdle muscular dystrophy type 2A or diabetes mellitus type 2. Their modulators may be useful in therapies of the above diseases [69]. Another enzyme, cyclooxygenase-2 (COX-2), is involved in arachidonic acid metabolism leading to the production of prostaglandin E_2_. Nonsteroidal anti-inflammatory drugs reveal anti-inflammatory and anti-tumor effects, especially via the inhibition of cyclooxygenase-2 activity [70,71]. To the best of our knowledge, there is no information about food peptides with similar activity.

The activity of the complement system, involving, e.g., proteolytic enzyme Complement factor B (alternative-complement-pathway C3/C5 convertase; EC 3.4.21.47) is an important part of the innate immunity [72]. However, this system’s function can exacerbate immune, inflammatory, and degenerative responses in pathological conditions, e.g., ischemic stroke [72]. Hyperactivation of the complement alternative pathway is associated with genetic and autoimmune diseases [73]. Compounds altering the action of Complement factor B may, thus, be classified as immunomodulating.

Neprilysin (EC 3.4.24.11) is a proteolytic enzyme involved in the metabolism of natriuretic peptides, angiotensin II, and many other endogenous bioactive peptides [74]. Neprilysin and its inhibitors are addressed in the research concerning the therapy of cardiovascular diseases, such as arterial hypertension [74] or chronic heart failure [75]. Oligopeptides being neprilysin inhibitors are annotated in the ChEMBL database.

Furin (EC 3.4.21.75) is a proteolytic enzyme cleaving many important proteins in mammalian (e.g., human) organisms. Receptors, hormones, growth factors, and cytokines are among its substrates. Its abnormal activity is associated with, e.g., cancers. Moreover, this enzyme cleaves some bacterial and viral proteins. Its aberrant activity also promotes infections [76].

Tyrosyl-tRNA synthetase (tyrosine–tRNA ligase; EC 6.1.1.1) is involved in protein biosynthesis and cell signaling. Products of its proteolysis stimulate blood vessel development as well as migration and activity of the immune system cells [77]. To the best of our knowledge, there is no information about food-derived peptides revealing interactions with this enzyme.

Neurotensin is a multifunctional neuropeptide. It is involved in the regulation of fat metabolism and appetite, but also pain, body temperature, learning, and memory. Its abnormal level is associated with, e.g., mood and eating disorders. Cognition decline associated with obesity is also supposed to be associated with abnormal neurotensin activity. The putative role of predicted ligands of neurotensin receptor 2 remains unclear [78].

Oligopeptide transporters are involved in the transport of oligopeptides and peptidomimetics [79]. Ligands of such proteins are expected to be easily absorbed from the digestive tract. Many peptides are annotated in ChEMBL as the ligands of a small intestine oligopeptide transporter isoform.

To summarize, the results of the prediction of peptide interactions using the SwissTargetPrediction program may serve as a guide for future research. The authors of this program recommend following such predictions by molecular docking and laboratory experiments with the most promising compounds [24]. On the other hand, we can emphasize that, apart from well-known targets for food peptides (ACE, DPP-IV), the predictions also included enzymes which so far had not been taken into account in the food and nutrition sciences. Many enzymes are inhibited by peptides as judged by the screening of chemical databases, such as ChEMBL [9]. Many peptides were known as ACE and DPP-IV inhibitors, but only a few of them acted as inhibitors of other enzymes [9]. The less-known peptide activities (from the food scientists’ point of view) are related to the enzymes which are addressed in the biological, medical, and pharmacological studies.

The prediction of drug-likeness and ADMET (absorption, distribution, metabolism, excretion, toxicity) properties of a molecule has recently become an obligatory step of in silico drug design [80,81]. Such properties also refer to bioactive food components, like peptides. Comparison of various properties of drugs and food components has recently become the focus of the scientific interests [82,83,84]. ADMET calculation would significantly aid the in silico evaluation of the potential bioactivity of peptides. To date, there were only few publications including the calculation of ADMET properties of bioactive peptides from food [85,86,87,88].

The most classic rule concerning the potential applicability of a compound as a drug (drug-likeness) is the so-called Rule of 5 [27]. A compound fulfilling the 5 has the molecular weight of up to 500 Da, a logarithm of octanol-water partition coefficient not exceeding 5, the number of hydrogen bond donors up to 5, and the number of hydrogen bond acceptors up to 10. Although the drug-likeness has recently not been considered obligatory to the pharmaceutical sciences, most of the existing drugs fulfill the above rule [89]. All ACE and DPP-IV inhibitors potentially released from collagens were subjected to this cheminformatic analysis, and all of the sequences fulfilled the Rule of 5 (see Table 5).

ADMET properties of peptides were interpreted based on the criteria presented and described on the website of the ADMETlab program [32]. Predicted Caco-2 permeability of peptides was low. According to the above criteria, the optimal logarithm of permeability should exceed −5.15. Three arginine-containing peptides had the lowest predicted logarithm of Caco-2 permeability (<−6.0). Caco-2 monolayers are recommended as models for simulation absorption of compounds from digestive tracts [90]. Seven out of the nine peptides revealed high predicted intestinal absorption probability (>0.3). Theoretical volume distribution (VD) values suggested that 8 peptides should be evenly distributed in tissues (VD within the range of 0.07–0.7 L × kg ^−1^). Peptide DR was predicted as confined to the blood (VD <0.07 L × kg ^−1^). It contains 4 ionizable groups, which make it strongly hydrophilic. More hydrophobic compounds can be evenly distributed or bound to the tissue compounds. Theoretical T1/2 (half-life time) values for peptides were very short. The calculated half-life time exceeding 1 h was observed only for RL peptide. According to the criteria described by Dong et al. [32], T1/2 for potential drugs is considered short if it does not exceed three hours. On the other hand, the compounds may reveal activity in vivo, and their half-life time can be longer than 0.5 h. Calculated LD_50_ values (in experimental work, LD_50_ is defined as a dose of a compound which kills 50% of tested animals) suggest low toxicity of peptides corresponding to 501–5000 mg × kg ^−1^. Thus, peptide RL can be considered non-toxic (LD_50_ > 5000 mg × kg ^−1^). In the case of peptides described here, results obtained from ADMETlab suggest that low Caco-2 permeability and short half-life time may limit the biological activity of oligopeptides in vivo. On the other hand, the predicted absorption probability of most of the peptides analyzed and low toxicity of all peptides should be their advantage.

## 4. Conclusions

Our protocol involving the quantitative parameters used to evaluate the potential of proteins to act as sources of biopeptides (A) and to release biopeptides due to the enzyme action (A_E_, W, and DH_t_) showed that collagens could be abundant in ACE- and DPP-IV-inhibiting peptides. To find out whether a protein can release peptides and which enzyme has an adequate potential to produce them, it is recommended to analyze those proteins for which A_E_ and W had relatively comparable values. Based on this, it was observed that pepsin and/or trypsin was an effective producer of ACE- and/or DPP-IV inhibitors during collagen hydrolysis. They were identified in vitro in other foods. However, their relatively high PeptideRanker Scores were not always indicative of their high bioactivity. Although our results give theoretical insights for further (i.e., laboratory) research, reliable results are dependent on continuous update of the database with the information regarding peptides, enzyme characteristics (specificity), and interpretation of the dataset. Considering the results of additional target predictions, we can conclude that the in silico prediction can discover lots of new information about interactions between food peptides and proteins (especially enzymes), even if a significant part of the results will be false-positive. ADMET prediction results are not fully conclusive. We can point out low toxicity as the advantage of biopeptides. On the other hand, the short predicted half-life time may limit their bioactivity. The methods from the area traditionally classified as chemical informatics, which are rather underutilized to date, may especially help enrich our knowledge about the bioactivity of food peptides, including those derived from collagens.

## Figures and Tables

**Table 1:** The frequency of the occurrence of peptides with a given activity (A) calculated for the collagens originating from different sources using the BIOPEP-UWM database tool (accessed: March 2020).

Source of Collagen	Major A (A ≥ 0.500)	Moderate A (A = 0.1002−0.499)	Minor A (A = 0.001−0.099)		
cow (*Bos taurus*)	0.834 ^ah 1^ 0.854 ^dpp^	0.214 ^am;re^ 0.238 ^at^	0.002 ^apr;^^35pd^	0.003 ^im^	0.006 ^ren^
			0.009 ^ne^	0.014 ^emb^	0.037 ^che^
			0.041 ^glui^	0.057 ^inh^	0.059 ^ao^
			0.069 ^st^	0.073 ^dpp3^	
pig (*Sus scrofa*)	0.846 ^dpp^ 0.847 ^ah^	0.216 ^am;re^ 0.238 ^at^	0.001 ^emb^	0.002 ^apr;35pd^	0.003 ^im^
			0.006 ^ren^	0.007 ^st^	0.008 ^ne^
			0.038 ^che^	0.042 ^glui^	0.058 ^inh^
			0.060 ^ao^	0.076 ^dpp3^	
sheep (*Ovis aries*)	0.833 ^ah^ 0.845 ^dpp^	0.198 ^am;re^ 0.215 ^at^	0.001 ^emb;im^	0.002 ^35pd^	0.003 ^apr^
			0.006 ^ren^	0.007 ^st^	0.008 ^ne^
			0.033 ^che^	0.045 ^glui^	0.052 ^inh^
			0.059 ^ao^	0.073 ^dpp3^	
chicken (*Gallus gallus*)	0.843 ^ah^ 0.852 ^dpp^	0.210 ^am;re^ 0.223 ^at^	0.001 ^lig^	0.002 ^apr;^^35pd;emb^	0.003 ^im^
			0.006 ^ren;is;st^	0.008 ^ne^	0.040 ^glui;che^
			0.057 ^inh^	0.061 ^ao^	0.075 ^dpp3^
duck (*Anas platyrhynchos platyrhynchos*)	0.847 ^ah^ 0.870 ^dpp^	0.240 ^at^ 0.210 ^am;re^	0.002 ^hypl;35pd^	0.003 ^lig^	0.004 ^emb^
			0.007 ^apr^	0.011 ^ren^	0.033 ^glui;che^
			0.046 ^inh^	0.057 ^ao^	0.083 ^dpp3^
horse (*Equus caballus*)	0.843 ^ah;dpp^	0.215 ^am;re^ 0.238 ^at^	0.001 ^emb^	0.002^35pd^	0.003 ^apr;im^
			0.006 ^ren^	0.007 ^st^	0.009 ^ne^
			0.037 ^che^	0.041 ^glui^	0.058 ^inh;ao^
			0.073 ^dpp3^		
salmon (*Salmo salar*)	0.798 ^dpp^ 0.799 ^ah^	0.170 ^at;am;re^	0.002 ^emb;is^	0.003^35pd;lig;apr^	0.005 ^st^
			0.008 ^ne;ren^	0.023 ^che^	0.029 ^glui^
			0.053 ^ao^	0.074 ^dpp3^	
rainbow trout (*Oncorhynchus mykiss*)	0.810 ^dpp^ 0.846 ^ah^	0.161 ^am^ 0.162 ^re^ 0.181 ^at^	0.001 ^hypl^	0.002 ^lig^	0.003 ^emb;35pd^
			0.006 ^apr^	0.008 ^st^	0.009 ^ne^
			0.013 ^ren^	0.015 ^glui^	0.018 ^che^
			0.020 ^inh^	0.034 ^ao^	0.071 ^dpp3^
goat (*Capra hircus*)	0.842 ^ah^ 0.849 ^dpp^	0.213 ^am;re^ 0.273 ^at^	0.001 ^emb^	0.002 ^apr;35pd^	0.003 ^im^
			0.006 ^ren^	0.007 ^st^	0.009 ^ne^
			0.037 ^che^	0.041 ^glui^	0.057 ^inh^
			0.058 ^ao^	0.074 ^dpp3^	
rabbit (*Oryctolagus cuniculus*)	0.834 ^ah^ 0.849 ^dpp^	0.199 ^am;re^ 0.215 ^at^	0.001 ^emb;im^	0.002 ^35pd^	0.003 ^apr^
			0.006 ^ren^	0.007 ^st^	0.008 ^ne^
			0.034 ^che^	0.045 ^glui^	0.052 ^inh^
			0.059 ^ao^	0.073 ^dpp3^	
turkey (*Meleagris gallopavo*)	0.822 ^dpp^ 0.841 ^ah^	0.192 ^am;re^ 0.220 ^at^	0.002 ^hypl^	0.003 ^lig;35pd^	0.004 ^emb^
			0.007 ^apr^	0.009 ^ne^	0.012 ^st^
			0.014 ^ren^	0.027 ^che^	0.030 ^glui^
			0.041 ^inh^	0.059 ^ao^	0.082 ^dpp3^

^1^ list of BIOPEP-UWM bioactivity codes of peptides: ^am^—antiamnestic, ^ah^—angiotensin I-converting enzyme (ACE, EC 3.4.15.1) inhibitor, im—immunomodulating, a^t^—antithrombotic, ^st^—stimulating, ^is^—immunostimulating, ^ne^—neuropeptide, ^re^—regulating, ^ao^—antioxidative, ^lig^—bacterial permease ligand, ^inh^—inhibitor, che—chemotactic, ^emb^—embryotoxic, ^apr^—activating ubiquitin-mediated proteolysis, ^dpp^—dipeptidyl peptidase IV (EC 3.4.14.5) inhibitor, ^glui^—α-glucosidase (EC 3.2.1.20) inhibitor, ^dpp3^—dipeptidyl peptidase (EC 3.4.14.4) III inhibitor, ^35pd^—calmodulin-dependent cyclic nucleotide phosphodiesterase (CaMPDE, EC 3.1.4.17) inhibitor, ^ren^—renin (EC 3.4.23.15) inhibitor, ^hypl^—hypolipidemic.

**Table 2:** Values of the BIOPEP-UWM parameters describing the computer simulation of proteolysis of collagens (accessed: March 2020).

Source of Collagen	Enzyme	A_E_	W	DH_t_ (%)
cow (*Bos taurus*)	B ^1^	0.158 ^dpp2^ 0.097 ^ah^ 0.042 ^am;at;re^ 0.014 ^dpp3^ 0.005 ^glui^ 0.001 ^ao^	0.25 ^im^ 0.195 ^am;re^ 0.191 ^dpp3^ 0.186 ^dpp^ 0.175 ^at^ 0.120 ^glui^ 0.115 ^ah^ 0.113 ^ren^ 0.024 ^ao^	55.55
	F	0.122 ^dpp^ 0.071 ^ah^ 0.021 ^am;at;re^ 0.008 ^dpp3^ 0.001 ^ao;ren^	0.226 ^ren^ 0.143 ^dpp^ 0.118 ^am;re^ 0.114 ^dpp3^ 0.105 ^at^ 0.084 ^ah^ 0.024 ^ao^	45.69
	Pap	0.151 ^dpp^ 0.105 ^ah^ 0.018 ^am;at;re^ 0.008 ^dpp3^ 0.001 ^ren^	1.000 ^lig^ 0.226 ^ren^ 0.176 ^dpp^ 0.124 ^ah^ 0.104 ^dpp3^ 0.084 ^am;re^ 0.076 ^at^ 0.012 ^ao^	46.11
	Pep	0.004 ^ah;dpp^ 0.002 ^ren^ 0.001 ^dpp3^	0.339 ^ren^ 0.019 ^dpp3^ 0.004 ^ah;dpp^	4.38
	T	0.002 ^ah^ 0.001 ^dpp;ao;at^	0.012 ^ao^ 0.006 ^at^ 0.003 ^ah^ 0.002 ^dpp^	8.75
	Pep + T	0.013 ^ah;dpp^ 0.005 ^dpp3^ 0.002 ^ren^ 0.001 ^at^	0.338 ^ren^ 0.067 ^dpp3^ 0.016 ^dpp^ 0.012 ^ao^ 0.006 ^at^	13.13
	Pep + T + Ch	0.141 ^ah^ 0.107 ^dpp^ 0.055 ^re;am;at^ 0.029 ^dpp3^ 0.005 ^ne^ 0.003 ^ren^ 0.002 ^ao^ 0.001 ^35pd^	0.667 ^35pd^ 0.544 ^ne^ 0.452 ^ren^ 0.409 ^dpp3^ 0.256 ^re;at^ 0.230 ^am^ 0.161 ^ah^ 0.125 ^dpp^ 0.036 ^ao^	45.63
pig (*Sus scrofa*)	B	0.158 ^dpp^ 0.098 ^ah^ 0.041 ^am;at;re^ 0.013 ^dpp3^ 0.006 ^glui^ 0.001 ^ao^	0.250 ^im^ 0.192 ^am;re^ 0.187 ^dpp^ 0.176 ^dpp3^ 0.174 ^at^ 0.133 ^glui^ 0.116 ^ah^ 0.111 ^ren^ 0.024 ^ao^	55.43
	F	0.124 ^dpp^ 0.721 ^ah^ 0.025 ^am;at;re^ 0.008 ^dpp3^ 0.001 ^ao;ren^	0.117 ^re^ 0.106 ^at^ 0.085 ^ah^ 0.117 ^am^ 0.024 ^ao^ 0.147 ^dpp^ 0.222 ^ren^ 0.102 ^dpp3^	45.91
	Pap	0.152 ^dpp^ 0.109 ^ah^ 0.018 ^am;at;re^ 0.007 ^dpp3^ 0.001 ^ren^	1.000 ^lig^ 0.222 ^ren^ 0.180 ^dpp^ 0.128 ^ah^ 0.093 ^dpp3^ 0.084 ^am;re^ 0.076 ^at^ 0.012 ^ao^	45.83
	Pep	0.003 ^ah;dpp^ 0.002 ^ren^	0.333 ^ren^ 0.009 ^dpp3^ 0.004 ^ah^ 0.003 ^dpp^	4.41
	T	0.001 ^at;ah^	0.012 ^ao^ 0.006 ^at^ 0.002 ^ah^	8.76
	Pep + T	0.013 ^ah^ 0.012 ^dpp^ 0.004 ^dpp3^ 0.002 ^ren^ 0.001 ^at^	0.333 ^ren^ 0.056 ^dpp3^ 0.015 ^ah^ 0.014 ^dpp^ 0.012 ^ao^ 0006 ^at^	13.17
	Pep + T + Ch	0.147 ^ah^ 0.109 ^dpp^ 0.055 ^am;at;re^ 0.031 ^dpp3^ 0.005 ^ne^ 0.003 ^ao;ren^ 0.001 ^35pd^	0.667 ^35pd^ 0.583 ^ne^ 0.444 ^ren^ 0.407 ^dpp3^ 0.256 ^am;re^ 0.232 ^at^ 0.174 ^ah^ 0.128 ^dpp^ 0.100 ^st^ 0.005 ^ao^	45.83
sheep (*Ovis aries*)	B	0.158 ^dpp^ 0.095 ^ah^ 0.041 ^am;at;re^ 0013 ^dpp3^ 0.005 ^glui^ 0.001 ^ao^	0.500 ^im^ 0.205 ^am;re^ 0.189 ^at^ 0.186 ^dpp^ 0.180 ^dpp3^ 0.114 ^ah^ 0.107 ^glui^ 0.023 ^ao^	55.69
	F	0.118 ^dpp^ 0.070 ^ah^ 0.023 ^am;at;re^ 0.007 ^dpp3^ 0.003 ^ao^	0.139 ^dpp^ 0.127 ^ren^ 0.115 ^am;re^ 0.106 ^at^ 0.095 ^dpp3^ 0.084 ^ah^ 0.047 ^ao^	45.69
	Pap	0.145 ^dpp^ 0.103 ^ah^ 0.019 ^am;at;re^ 0.006 ^dpp3^	1.000 ^lig^ 0.175 ^dpp^ 0.127 ^ren^ 0.124 ^ah^ 0.094 ^am;re^ 0.087 ^at^ 0.075 ^dpp3^ 0.016 ^glui^ 0.012 ^ao^	45.62
	Pep	0.003 ^ah;dpp^ 0.001 ^dpp3;ren^	0.255 ^ren^ 0.019 ^dpp3^ 0.004 ^ah^ 0.003 ^dpp^	4.55
	T	0.001 ^ah^	0.012 ^ao^ 0.003 ^at^ 0.002 ^ah^	9.10
	Pep + T	0.012 ^ah;dpp^ 0.005 ^dpp3^ 0.001 ^ren^	0.255 ^ren^ 0.066 ^dpp3^ 0.015 ^ah^ 0.014 ^dpp^ 0.012 ^ao^ 0.003 ^at^	13.65
	Pep + T + Ch	0.137 ^ah^ 0.107 ^dpp^ 0.050 ^am;at;re^ 0.030 ^dpp3^ 0.004 ^ne^ 0.003 ^ren^ 0.002 ^ao^ 0.001 ^35pd^	0.667 ^35pd^ 0.509 ^ren^ 0.494 ^ne^ 0.410 ^dpp3^ 0.250 ^am;re^ 0.231 ^at^ 0.165 ^ah^ 0.127 ^dpp^ 0.101 ^st^ 0.036 ^ao^	46.04
chicken (*Gallus gallus*)	B	0.154 ^dpp^ 0.096 ^ah^ 0.043 ^am;at,re^ 0.012 ^dpp3^ 0.006 ^glui^ 0.001 ^ao;im^	0.500 ^im^ 0.203 ^am;re^ 0.185 ^at^ 0.180 ^dpp^ 0.159 ^dpp3^ 0.141 ^glui^ 0.114 ^ah^ 0.111 ^ren^ 0.023 ^ao^	55.59
	F	0.120 ^dpp^ 0.068 ^ah^ 0.025 ^am;at;re^ 0.008 ^dpp3^ 0.003 ^ao^ 0.001 ^ren^	0.222 ^ren^ 0.141 ^dpp^ 0.116 ^am;re^ 0.107 ^at^ 0.103 ^dpp3^ 0.080 ^ah^ 0.047 ^ao^	44.97
	Pap	0.158 ^dpp^ 0.108 ^ah^ 0.019 ^am;at;re^ 0.007 ^dpp3^ 0.001 ^ren^	0.500 ^lig^ 0.250 ^im^ 0.222 ^ren^ 0.185 ^dpp^ 0.128 ^ah^ 0.094 ^dpp3^ 0.090 ^am;re^ 0.082 ^at^ 0.012 ^ao^	46.43
	Pep	0.003 ^ah;dpp^ 0.002 ^ren^	0.333 ^ren^ 0.009 ^dpp3^ 0.003 ^ah;dpp^	4.55
	T	0.002 ^ah^ 0.001 ^dpp^	0.012 ^ao^ 0.003 ^ah;at^ 0.001 ^dpp^	8.60
	Pep + T	0.013 ^ah;dpp^ 0.004 ^dpp3^ 0.002 ^ren^	0.333 ^ren^ 0.056 ^dpp3^ 0.016 ^ah^ 0.012 ^ao^ 0.003 ^at^	13.15
	Pep + T + Ch	0.143 ^ah^ 0.112 ^dpp^ 0.055 ^am;at;re^ 0.033 ^dpp3^ 0.005 ^ao^ 0.004 ^ne^ 0.003 ^ren^ 0.001 ^35pd^	0.667 ^35pd^ 0.500 ^ne^ 0.444 ^ren^ 0.439 ^dpp3^ 0.259 ^am;re^ 0.237 ^at^ 0.170 ^ah^ 0.131 ^dpp^ 0.125 ^st^ 0.082 ^ao^	45.38
duck (*Anas platyrhynchos platyrhynchos*)	B	0.146 ^dpp^ 0.091 ^ah^ 0.034 ^am;at;re^ 0.015 ^dpp3^ 0.005 ^ren^ 0.003 ^glui^ 0.002 ^ao; st^ 0.001 ^35pd^	0.652 ^35pd^ 0.533 ^hyp^ 0.397 ^ren^ 0.247 ^st^ 0.178 ^dpp3^ 0.172 ^dpp^ 0.163 ^am;re^ 0.144 ^at^ 0.104 ^ah^ 0.096 ^glui^ 0.041 ^ao^	57.79
	F	0.123 ^dpp^ 0.083 ^ah^ 0.025 ^am;at;re^ 0.012 ^dpp3^ 0.005 ^ren^ 0.002 ^st^ 0.001 ^ao; hyp,35pd^	0.533 ^hyp^ 0.397 ^ren^ 0.348 ^35pd^ 0.248 ^st^ 0.146 ^dpp^ 0.130 ^dpp3^ 0.126 ^re^ 0.119 ^am^ 0.105 ^at^ 0.095 ^ah^ 0.014 ^ao^	48.57
	Pap	0.154 ^dpp^ 0.113 ^ah^ 0.019 ^am;at;re^ 0.010 ^dpp3^ 0.004 ^ren^ 0.002 ^ao; st^ 0.001 ^hyp;lig;35pd^	0.533 ^hyp^ 0.348 ^35pd^ 0.336 ^ren^ 0.258 ^lig^ 0.182 ^dpp^ 0.161 ^st^ 0.130 ^ah^ 0.122 ^dpp3^ 0.092 ^am;re^ 0.082 ^at^ 0.027 ^ao^	47.87
	Pep	0.005 ^ah^ 0.004 ^dpp^ 0.001 ^dpp3;ren^	0.070 ^ren^ 0.010 ^dpp3^ 0.006 ^ah^ 0.005 ^dpp^	5.58
	T	0.001 ^ah;at;dpp^	0.034 ^at^ 0.001 ^ah;dpp^	8.83
	Pep + T	0.013 ^ah;dpp^ 0.002 ^at;dpp3^ 0.001 ^ren^	0.070 ^ren^ 0.018 ^dpp3^ 0.016 ^dpp^ 0.015 ^ah^ 0.006 ^at^	14.41
	Pep + T + Ch	0.024 ^dpp^ 0.023 ^ah^ 0.003 ^at^ 0.001 ^ao;dpp3;glui;reg;ren^	0.070 ^ren^ 0.028 ^dpp^ 0.027 ^ah;ao^ 0.025 ^glui^ 0.013 ^at^ 0.010 ^dpp^_3_ 0.004 ^re^	20.99
horse (*Equus caballus*)	B	0.155 ^dpp^ 0.096 ^ah^ 0.042 ^am;at;re^ 0.013 ^dpp3^ 0.006 ^glui^ 0.001 ^ao;im;ren^	0.25 ^im^ 0.193 ^am;re^ 0.184 ^dpp^ 0.181 ^dpp3^ 0.175 ^at^ 0.115 ^glui^ 0.114 ^ah^ 0.113 ^ren^ 0.024 ^ao^	55.55
	F	0.124 ^dpp^ 0.074 ^ah^ 0.026 ^am;at;re^ 0.008 ^dpp3^ 0.001 ^ao;ren^	0.226 ^ren^ 0.147 ^dpp^ 0.123 ^am;re^ 0.112 ^at^ 0.104 ^dpp3^ 0.088 ^ah^ 0.012 ^ao^	45.83
	Pap	0.151 ^dpp^ 0.108 ^ah^ 0.018 ^am;at;re^ 0.007 ^dpp3^ 0.001 ^ao;lig;ren^	1.000 ^lig^ 0.226 ^ren^ 0.179 ^dpp^ 0.128 ^ah^ 0.095 ^dpp3^ 0.084 ^am;re^ 0.077 ^at^ 0.012 ^ao^	45.90
	Pep	0.004 ^ah^ 0.003 ^dpp^ 0.021 ^ren^ 0.001 ^dpp3^	0.339 ^ren^ 0.010 ^dpp3^ 0.004 ^ah^ 0.003 ^dpp^	4.31
	T	0.001 ^dpp;ah;ao,at^	0.012 ^ao^ 0.006 ^at^ 0.002 ^ah^ 0.001 ^dpp^	8.82
	Pep + T	0.013 ^ah^ 0.012 ^dpp^ 0.004 ^dpp3^ 0.002 ^ren^ 0.001 ^ao;at^	0.339 ^ren^ 0.058 ^dpp3^ 0.015 ^ah^ 0.014 ^dpp^ 0.012 ^ao^ 0.006 ^at^	13.13
	Pep + T + Ch	0.022 ^dpp^ 0.019 ^ah^ 0.005 ^dpp3^ 0.003 ^at;ren^ 0.002 ^ao^ 0.001 ^glui; st;35pd^	0.452 ^ren^ 0.333 ^35pd^ 0.101 ^st^ 0.067 ^dpp3^ 0.036 ^ao^ 0.026 ^dpp^ 0.023 ^ah^ 0.014 ^glui^ 0.012 ^at^	17.85
salmon (*Salmo salar*)	B	0.143 ^dpp^ 0.093 ^ah^ 0.036 ^am;at;re^ 0.008 ^glui^ 0.006 ^dpp3^ 0.003 ^ren^ 0.002 ^ao^ 0.001 ^st;35pd^	0.333 ^ren^ 0.250 ^35pd^ 0.223 ^glui^ 0.219 ^at^ 0.218 ^re^ 0.198 ^at^ 0.179 ^dpp^ 0.143 ^st^ 0.116 ^ah^ 0.086 ^dpp3^ 0.040 ^ao^	57.85
	F	0.115 ^dpp^ 0.071 ^ah^ 0.023 ^am;at;re^ 0.006 ^dpp3^ 0.004 ^ao;ren^ 0.001 ^HMGi;35pd^	1.000 ^HMGi^ 0.417 ^ren^ 0.250 ^35pd^ 0.144 ^dpp^ 0.139 ^am^ 0.139 ^re^ 0.126 ^at^ 0.089 ^ah^ 0.086 ^dpp3^ 0.067 ^ao^	47.48
	Pap	0.144 ^dpp^ 0.110 ^ah^ 0.0203 ^am;at;re^ 0.006 ^dpp3^ 0.003 ^ren^ 0.001 ^ao;HMGi;35pd^	1.000 ^HMGi^ 0.333 ^ren^ 0.250 ^35pd^ 0.181 ^dpp^ 0.138 ^ah^ 0.122 ^am;re^ 0.112 ^at^ 0.076 ^dpp3^ 0.027 ^ao^	46.91
	Pep	0.003 ^ah^ 0.002 ^dpp^ 0.001 ^ao;dpp3;ren^	0.167 ^ren^ 0.013 ^ao^ 0.010 ^dpp3^ 0.003 ^ah;dpp^	4.07
	T	0.002 ^ah^ 0.001 ^dpp^	0.003 ^ah^ 0.002 ^dpp^	8.77
	Pep + T	0.010 ^dpp^ 0.009 ^ah^ 0.006 ^dpp3^ 0.001 ^ren^	0.167 ^ren^ 0.076 ^dpp3^ 0.012 ^dpp^ 0.001 ^ah^	12.83
	Pep + T + Ch	0.019 ^ah^ 0.018 ^dpp^ 0.008 ^dpp3^ 0.003 ^ao^ 0.002 ^at;ren^ 0.001 ^glui; is;35pd^	0.333 ^is^ 0.250 ^ren;35pd^ 0.105 ^dpp3^ 0.053 ^ao^ 0.024 ^ah^ 0.023 ^dpp^ 0.021 ^glui^ 0.011 ^at^	18.93
rainbow trout (*Oncorhynchus mykiss*)	B	0.142 ^dpp^ 0.096 ^ah^ 0.034 ^am;at;re^ 0.013 ^dpp3^ 0.003 ^ao;glui;ren^ 0.002 ^35pd^ 0.001 ^hyp; st^	0.500 ^35pd^ 0.467 ^hyp^ 0.236 ^ren^ 0.209 ^am^ 0.208 ^re^ 0.187 ^at^ 0.178 ^dpp3^ 0.143 ^glui^ 0.113 ^ah^ 0.093 ^st^ 0.082 ^ao^	61.89
	F	0.118 ^dpp^ 0.098 ^ah^ 0.027 ^am;at;re^ 0.008 ^dpp3^ 0.003 ^ao^ 0.002 ^ren^ 0.001 ^HMGi; st;35pd^	1.000 ^HMGi^ 0.333 ^35pd^ 0.173 ^ren^ 0.171 ^re^ 0.168 ^am^ 0.149 ^at^ 0.147 ^dpp^ 0.115 ^ah;dpp3^ 0.093 ^st^ 0.082 ^ao^	52.44
	Pap	0.153 ^dpp^ 0.137 ^ah^ 0.025 ^am;at;re^ 0.008 ^dpp3^ 0.004 ^ren^ 0.002 ^ao;glui;35pd^ 0.001 ^HMGi; hyp; st;lig^	1.000 ^HMGi^ 0.500 ^35pd^ 0.467 ^hyp^ 0.318 ^lig^ 0.291 ^ren^ 0.190 ^dpp^ 0.162 ^ah^ 0.153 ^am;re^ 0.138 ^at^ 0.115 ^dpp3^ 0.105 ^glui^ 0.093 ^st^ 0.060 ^ao^	51.46
	Pep	0.003 ^ah;dpp^ 0.001 ^dpp3;ren^	0.055 ^ren^ 0.010 ^dpp3^ 0.004 ^ah;dpp^	5.48
	T	0.002 ^at^ 0.001 ^ah^	0.008 ^at^ 0.001 ^ah^	8.93
	Pep + T	0.011 ^ah;dpp^ 0.002 ^at;dpp3^ 0.001 ^ren^	0.055 ^ren^ 0.031 ^dpp3^ 0.014 ^dpp^ 0.013 ^ah^ 0.008 ^at^	14.40
	Pep + T + Ch	0.022 ^ah^ 0.020 ^dpp^ 0.002 ^ao;at;dpp3^ 0.001 ^glui; st;re;ren^	0.093 ^st^ 0.060 ^ao;ren^ 0.033 ^glui^ 0.026 ^ah^ 0.021 ^dpp3^ 0.008 ^at^ 0.004 ^re^	21.61
go ^at^ (*Capra hircus*)	B	0.156 ^dpp^ 0.096 ^ah^ 0.042 ^am;at;re^ 0.014 ^dpp3^ 0.005 ^glui^ 0.001 ^ao;im;ren^	0.250 ^im^ 0.195 ^am;re^ 0.189 ^dpp3^ 0.184 ^dpp^ 0.175 ^at^ 0.114 ^ah^ 0.113 ^ren^ 0.104 ^glui^ 0.024 ^ao^	55.55
	F	0.120 ^dpp^ 0.069 ^ah^ 0.024 ^am;at;re^ 0.008 ^dpp3^ 0.001 ^ao;ren^	0.226 ^ren^ 0.114 ^dpp^ 0.113 ^dpp3^ 0.111 ^am;re^ 0.010 ^at^ 0.082 ^ah^ 0.024 ^ao^	45.55
	Pap	0.152 ^dpp^ 0.105 ^ah^ 0.019 ^am;at;re^ 0.008 ^dpp3^ 0.001 ^ao;lig;ren^	1.000 ^lig^ 0.226 ^ren^ 0.179 ^dpp^ 0.125 ^ah^ 0.103 ^dpp3^ 0.088 ^am;re^ 0.079 ^at^ 0.012 ^ao^	46.18
	Pep	0.040 ^ah;dpp^ 0.002 ^ren^ 0.001 ^dpp3^	0.339 ^ren^ 0.019 ^dpp3^ 0.004 ^ah;dpp^	4.38
	T	0.01 ^ah;ao;at;dpp^	0.117 ^ao^ 0.006 ^at^ 0.02 ^ah^ 0.001 ^dpp^	8.75
	Pep + T	0.013 ^ah;dpp^ 0.005 ^dpp3^ 0.002 ^ren^ 0.001 ^ao;at^	0.339 ^ren^ 0.067 ^dpp3^ 0.015 ^ah;dpp^ 0.012 ^ao^ 0.006 ^at^	13.13
	Pep + T + Ch	0.023 ^dpp^ 0.021 ^ah^ 0.006 ^dpp3^ 0.003 ^am;at;re^ 0.001 ^glui; st;35pd^	0.452 ^ren^ 0.333 ^35pd^ 0.101 ^st^ 0.008 ^dpp3^ 0.045 ^ao^ 0.027 ^dpp^ 0.025 ^ah^ 0.015 ^glui^ 0.012 ^at^	18.06
rabbit (*Oryctolagus cuniculus*)	B	0.158 ^dpp^ 0.095 ^ah^ 0.041 ^am;at;re^ 0.013 ^dpp3^ 0.005 ^glui^ 0.001 ^ao;im^	0.500 ^im^ 0.205 ^am;re^ 0.189 ^at^ 0.186 ^dpp^ 0.179 ^dpp3^ 0.114 ^ah^ 0.097 ^glui^ 0.024 ^ao^	55.72
	F	0.118 ^dpp^ 0.070 ^ah^ 0.023 ^am;at;re^ 0.007 ^dpp3^ 0.003 ^ao^ 0.001 ^ren^	0.134 ^dpp^ 0.127 ^ren^ 0.114 ^am;re^ 0.106 ^at^ 0.094 ^dpp3^ 0.084 ^ah^ 0.047 ^ao^	45.72
	Pap	0.149 ^dpp^ 0.103 ^ah^ 0.019 ^am;at;re^ 0.006 ^dpp3^ 0.001 ^ao;glui;lig;ren^	1.000 ^lig^ 0.175 ^dpp^ 0.127 ^ren^ 0.124 ^ah^ 0.094 ^am;re^ 0.087 ^at^ 0.075 ^dpp3^ 0.014 ^glui^ 0.012 ^ao^	45.66
	Pep	0.003 ^ah;dpp^ 0.001 ^ren;dpp3^	0.255 ^ren^ 0.019 ^dpp3^ 0.004 ^ah^ 0.003 ^dpp^	4.55
	T	0.001 ^ah;ao;at;dpp^	0.012 ^ao^ 0.003 ^at^ 0.002 ^at^ 0.001 ^dpp^	9.10
	Pep + T	0.012 ^ah;dpp^ 0.005 ^dpp3^ 0.001 ^ao;at;ren^	0.255 ^ren^ 0.066 ^dpp3^ 0.015 ^ah^ 0.014 ^dpp^ 0.012 ^ao^ 0.003 ^at^	13.66
	Pep + T + Ch	0.021 ^dpp^ 0.020 ^ah^ 0.006 ^dpp3^ 0.003 ^ao^ 0.002 ^at;ren^ 0.001 ^glui; st;35pd^	0.382 ^ren^ 0.333 ^35pd^ 0.101 ^st^ 0.075 ^dpp3^ 0.057 ^ao^ 0.025 ^dpp^ 0.024 ^ah^ 0.014 ^glui^ 0.001 ^at^	18.83
turkey (*Meleagris gallopavo*)	B	0.141 ^dpp^ 0.092 ^ah^ 0.032 ^am;at;re^ 0.016 ^dpp3^ 0.005 ^ren^ 0.004 ^ao^ 0.003 ^glui^ 0.002 ^st;35pd^ 0.001 ^hyp^	0.767 ^35pd^ 0.533 ^hyp^ 0.387 ^ren^ 0.196 ^dpp3^ 0.166 ^am;re^ 0.146 ^at^ 0.110 ^ah^ 0.080 ^glui^ 0.065 ^ao^	57.74
	F	0.118 ^dpp^ 0.082 ^ah^ 0.022 ^re^ 0.021 ^am;at^ 0.011 ^dpp3^ 0.005 ^ren^ 0.003 ^st^ 0.002 ^ao^ 0.001 ^hyp;35pd^	0.533 ^hyp^ 0.500 ^35pd^ 0.336 ^ren^ 0.246 ^st^ 0.144 ^dpp^ 0.131 ^dpp3^ 0.115 ^reg^ 0.120 ^am^ 0.098 ^ah^ 0.097 ^at^ 0.039 ^ao^	48.82
	Pap	0.147 ^dpp^ 0.110 ^ah^ 0.019 ^am;at;re^ 0.011 ^dpp3^ 0.005 ^ren^ 0.003 ^ao^ 0.002 ^glui; st;35pd^ 0.001 ^hyp;lig^	0.533 ^hyp^ 0.500 ^35pd^ 0.336 ^ren^ 0.267 ^lig^ 0.179 ^dpp^ 0.131 ^ah;dpp3^ 0.123 ^st^ 0.100 ^re^ 0.087 ^at^ 0.087 ^glui^ 0051 ^ao^	47.52
	Pep	0.006 ^ah^ 0.005 ^dpp^ 0.001 ^ao;ren;dpp3^	0.058 ^ren^ 0.018 ^dpp3^ 0.014 ^ao^ 0.007 ^ah^ 0.006 ^dpp^	6.18
	T	0.002 ^ah^ 0.001 ^dpp^	0.003 ^ah^ 0.002 ^dpp^	9.15
	Pep + T	0.017 ^dpp^ 0.016 ^ah^ 0.003 ^dpp3^ 0.001 ^ao;at;ren^	0.058 ^ren^ 0.037 ^dpp3^ 0.204 ^dpp^ 0.019 ^ah^ 0.014 ^ao^ 0.004 ^at^	15.33
	Pep + T + Ch	0.136 ^ah^ 0.106 ^dpp^ 0.047 ^re^ 0.046 ^am;at^ 0.030 ^dpp3^ 0.003 ^ne^ 0.002 ^ao^ 0.001 ^ren; st^	0.364 ^dpp3^ 0.330 ^ne^ 0.241 ^re^ 0.238 ^am^ 0.208 ^at^ 0.161 ^ah^ 0.129 ^dpp^ 0.066 ^st^ 0.058 ^ren^ 0039 ^ao^	45.69

^1^ B-bromelain, Ch-chymotrypsin, F-ficin, Pap-papain, Pep-pepsin, T-trypsin. ^2^ list of BIOPEP-UWM bioactivity codes of peptides: ^am^—antiamnestic, ^ah^—ACE (EC 3.4.15.1) inhibitor, ^im^—immunomodulating, ^at^—antithrombotic, ^st^—stimulating, ^is^—immunostimulating, ^ne^—neuropeptide, ^re^—regulating, ^ao^—antioxidative, ^lig^—bacterial permease ligand, ^inh^—inhibitor, ^che^—chemotactic, ^emb^—embryotoxic, ^apr^—activating ubiquitin mediated proteolysis, ^dpp^—dipeptidyl peptidase IV (EC 3.4.14.5) inhibitor, ^glui^—α-glucosidase (EC 3.2.1.20) inhibitor, ^dpp3^—dipeptidyl peptidase III (EC 3.4.14.4) inhibitor, ^35pd^—CaMPDE (EC 3.1.4.17) inhibitor, ^ren^—renin (EC 3.4.23.15) inhibitor, ^hypl^—hypolipidemic, ^HMGi^—3-hydroxy-3-methyl-glutaryl-CoA (HMG-CoA) reductase (EC 1.1.1.34) inhibitor.

**Table 3:** ACE and DPP-IV inhibitors potentially produced from collagens (data retrieved from BIOPEP-UWM database; accessed: April 2020).

Peptide Sequence	Peptideranker Score	Collagen Source	Enzyme Applied
GF ^ACEi;DPP-IVi 1^	0.994	cow (*Bos Taurus)*/sheep (*Ovis aries*)	Pep ^2^
SF^ACEi;DPP-IVi^	0.948	cow (*Bos taurus*)/pig (*Sus scrofa*)/sheep (*Ovis aries*)/chicken (*Gallus gallus*)/horse (*Equus caballus*)	Pep
QF ^DPP-IVi^	0.946	cow (*Bos taurus*)/chicken (*Gallus gallus*)/	Pep
DF ^ACEi^	0.942	horse (*Equus caballus)*	Pep
PGL ^ACEi^	0.855	cow (*Bos taurus*)/pig (*Sus scrofa*)/chicken (*Gallus gallus*)/horse (*Equus caballus*)/**salmon (*Salmo salar*) ^3^**	Pep
TF ^ACEi;DPP-IVi^	0.826	cow (*Bos taurus*)/pig (*Sus scrofa*)/sheep (*Ovis aries*)/chicken (*Gallus gallus*)/horse (*Equus caballus*)/salmon (*Salmo salar*)	Pep
GR ^ACEi^	0.766	rainbow trout (*Oncorhynchus mykiss*)	T ^4^
RL ^ACEi;DPP-IVi 4^	0.626	cow (*Bos taurus*)/pig (*Sus scrofa*)/sheep (*Ovis aries*)/chicken (*Gallus gallus*)/horse (*Equus caballus*)/salmon (*Salmo salar)*	Pep
DR ^DPP-IVi^	0.289	horse (*Equus caballus*)	T

^1^ ACE_i_ and DPP-IV_i_—angiotensin converting enzyme inhibitor and dipeptidyl peptidase IV inhibitor, respectively, ^2^ Pep—pepsin, ^3^ **bold font**—collagen hydrolysate source in which the peptide was identified more than once; ^4^ T—trypsin.

**Table 4:** Top-ranked human proteins predicted to be the targets for peptides potentially released from collagens (see Table 3) (data retrieved from SwissTargetPrediction web-tool; accessed: May 2020).

Peptide Sequence	Protein 1	Protein 2	Protein 3
PGL	Dipeptidyl peptidase IV (UniProt—P27487 ^1^; ChEMBL—CHEMBL284 ^2^) Probability: 0.526 ^3^	Angiotensin converting enzyme (UniProt—P12821; ChEMBL—CHEMBL1808) Probability: 0.445	Cyclooxygenase-2 (UniProt—P35354; ChEMBL—CHEMBL230) Probability: 0.420
RL	Neurotensin receptor 2 (UNiProt—O95665; ChEMBL—CHEMBL2514) Probability: 0.166	Complement factor B (UniProt—P00751; ChEMBL—CHEMBL573) Probability: 0.166	Subtilisin/kexin type 6 (UniProt—P29122; ChEMBL—CHEMBL2951) Probability: 0.133
GF	Oligopeptide transporter small intestine isoform (UniProt—P46059; ChEMBL—CHEMBL4605) Probability: 0.130	Calpain 1 (UniProt—P07384; ChEMBL—CHEMBL389) Probability: 0.112	Neprilysin (UniProt—P08473; ChEMBL—CHEMBL1944) Probability: 0.104
SF	Calpain 1 (UniProt—P07384; ChEMBL—CHEMBL3891) Probability: 0.081	Oligopeptide transporter small intestine isoform (UniProt—P46059; ChEMBL—CHEMBL4605) Probability: 0.072	Cyclooxygenase-2 (UniProt—P35354; ChEMBL—CHEMBL230) Probability: 0.063
TF	Calpain 1 (UniProt—P07384; ChEMBL—CHEMBL3891) Probability: 0.238	Tyrosyl-tRNA synthetase (UniProt—P54577; ChEMBL—CHEMBL3179) Probability: 0.143	Cyclooxygenase-2 (UniProt—P35354; ChEMBL—CHEMBL230) Probability: 0.143
QF	Angiotensin converting enzyme (UniProt—P12821; ChEMBL—CHEMBL1808) Probablity: 0.238	Calpain 1 (UniProt—P07384; ChEMBL—CHEMBL3891) Probability: 0.230	Tyrosyl-tRNA synthetase (UniProt—P54577; ChEMBL—CHEMBL3179) Probability: 0.140
DF	Calpain 1 (UniProt—P07384; ChEMBL—CHEMBL3891) Probability: 0.150	Angiotensin converting enzyme (UniProt—P12821; ChEMBL—CHEMBL1808) Probablity: 0.117	Neprilysin (UniProt—P08473; ChEMBL—CHEMBL1944) Probability: 0.109
DR	Complement factor B (UniProt—P00751; ChEMBL—CHEMBL5731) Probability: 0.109	Furin (UniProt—P09958; ChEMBL—CHEMBL2611) Probability: 0.109	Integrin alpha-IIb/beta-3 (UniProt—P08514; P05106; ChEMBL—CHEMBL2093869) Probability: 0.109
GR	Complement factor B (UniProt—P00751; ChEMBL—CHEMBL5731) Probability: 0.112	Furin (UniProt—P09958; ChEMBL—CHEMBL2611) Probability: 0.104	Neurotensin receptor 2 (UNiProt—O95665; ChEMBL—CHEMBL2514) Probability: 0.086

^1^ UniProt database accession numbers, ^2^ ChEMBL database ID numbers (https://www.ebi.ac.uk/chembl/) [66], ^3^ probability of the peptide to be a ligand of a given protein.

**Table 5:** Predicted ADMET (absorption, distribution, metabolism, excretion, toxicity) properties of the ACE- and DPP-IV-inhibiting peptides potentially produced from collagens (data retrieved from ADMETlab; accessed: May 2020).

Sequence	Rule of 5	Log Caco-2 Permeability (Permeability Expressed in cm × s ^−1^)	Human Intestinal Absorption Probability	VD ^1^ (L × kg ^−1^)	T 1/2 ^2^ (h)	LD_50_^3^ of Acute Toxicity (mg × kg ^−1^)
PGL	+	−5.643	0.309	0.149	0.701	1589
RL	+	−6.203	0.398	0.160	1.184	45,963
GF	+	−5.354	0.482	0.209	0.691	1344
SF	+	−5.818	0.281	0.130	0.663	1513
TF	+	−5.781	0.310	0.103	0.660	1385
QF	+	−5.929	0.368	0.090	0.578	1592
DF	+	−5.625	0.385	0.072	0.580	1672
DR	+	−6.407	0.275	0.054	0.811	1494
GR	+	−6.292	0.335	0.150	0.962	1140

^1^ VD—volume distribution, ^2^ T1/2—theoretical half -life time, ^3^ LD_50_—dose of a compound which kills 50% of tested animals.

## Supplementary Materials

The following are available online at https://www.mdpi.com/2304-8158/9/7/965/s1. **Table S1**. SMILES strings and structures of peptides with ionized acidic and basic groups. **Table S2**. Predicted targets for PGL peptide. Red font indicates 15 most likely targets. **Table S3**. Predicted targets for RL peptide. Red font indicates 15 most likely targets. **Table S4**. Predicted targets for GF peptide. Red font indicates 15 most likely targets. **Table S5**. Predicted targets for SF peptide. Red font indicates 15 most likely targets. **Table S6**. Predicted targets for TF peptide. Red font indicates 15 most likely targets. **Table S7**. Predicted targets for QF peptide. Red font indicates 15 most likely targets. **Table S8**. Predicted targets for DF peptide. Red font indicates 15 most likely targets. **Table S9**. Predicted targets for DR peptide. Red font indicates 15 most likely targets. **Table S10**. Predicted targets for GR peptide. Red font indicates 15 most likely targets.

## Abbreviations

ACE	angiotensin converting enzyme (EC 3.4.15.1)
ACE_i_	angiotensin converting enzyme inhibitor
ADMET	absorption, distribution, metabolism, excretion, toxicity
BIOPEP-UWM	database of protein and bioactive peptide sequences (http://www.uwm.edu.pl/biochemia) [22]
A	depending on the context: alanine or the frequency of the occurrence of bioactive fragments in a protein sequence [22] described by the following equation: A = a/N where a—the number of fragments with a given activity, N—the number of amino acid residues in a protein
A_E_	The frequency of release of fragments with a given activity by selected enzymes [22,23] described by the following equation: A_E_ = d/N where d-the number of peptides with a given activity (e.g., ACE inhibitors) released by a given enzyme (e.g., trypsin) N-the number of amino acid residues in a protein
B	stem bromelain (bromelain) (EC 3.4.22.32)
CaMPDE	calmodulin-dependent cyclic nucleotide phosphodiesterase (EC 3.1.4.17)
CH	collagen hydrolysate
Ch	chymotrypsin (EC 3.4.21.1)
ChEMBL	ChEMBL database of molecules with drug-like properties (https://www.ebi.ac.uk/chembl) [66]
CoA	coenzyme A
Complement factor B	alternative-complement-pathway C3/C5 convertase (EC 3.4.21.47)
COX-2	cyclooxygenase-2 (prostaglandin-endoperoxide synthase; EC 1.14.99.1)
DH_t_	theoretical degree of hydrolysis (%) [22] described by the following equation: DH_t_ = (d/D) × 100% where d—the number of hydrolyzed peptide bonds in a protein/peptide chain D—the total number of peptide bonds in a protein/peptide chain
DPP-III	dipeptidyl peptidase III (EC 3.4.14.4)
DPP-IV	dipeptidyl peptidase IV (EC 3.4.14.5)
DPP-IV_i_	dipeptidyl peptidase IV inhibitor
F	depending on the context: phenylalanine or ficin (EC 3.4.22.3)
G	glycine
HMG-CoA	3-hydroxy-3-methyl-glutaryl-CoA reductase (EC 1.1.1.34)
HT	high throughput technology
Hyp	hydroxyl-proline/hydroxyl-lysine
IC_50_	concentration of a peptide corresponding to its half-inhibitory effect (μM)
IUBMB	International Union of Biochemistry and Molecular Biology
LD_50_	dose of a compound which kills 50% tested animals (mg × kg ^−1^)
P	proline
Pap	papain (EC 3.4.22.2)
Pep	pepsin (EC 3.4.23.1)
QSAR	Quantitative Structure-Activity Relationship [19]
SMILES	Simplified Molecular Input Line Entry Specification [25]
T	depending on the context: treonine or trypsin (EC 3.4.21.4)
T1/2	theoretical half-life time (h)
VD	volume distribution (L × kg ^−1^)
W	depending on the context: tryptophan or the relative frequency of release of fragments with a given activity by selected enzymes [22] described by the following equation: W = A_E_/A where A_E_—the frequency of release of fragments with a given activity by selected enzymes (see above) A—the frequency of bioactive fragments occurrence in a protein sequence (see above).
