# Supplementary material for: Characteristics of Biopeptides Released In Silico from Collagens Using Quantitative Parameters
Source: Foods. 2020 Jul 21;9(7):965. doi: 10.3390/foods9070965 (PMC7404701; doi:10.3390/foods9070965)
Supplement: Supplementary file 1 [file foods-09-00965-s001.pdf]

Characteristics of biopeptides released *in silico* from collagens using quantitative parameters

Anna Iwaniak*, Piotr Minkiewicz, Monika Pliszka, Damir Mogut, Małgorzata Darewicz

Supplement

Table S1. SMILES strings and structures of peptides with ionized acidic and basic groups.

Sequence	BIOPEP-UWM ID[1]	SMILES[2]	Structure
PGL	7507	[H][C@@](CC(C)C)(NC(=O)CNC(=O)[C@]1([H])CCC[NH2+]1)C([O-])=O	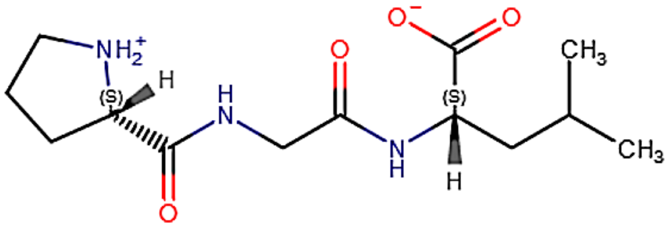
RL	3257; 8886	[H][C@]([NH3+])(CCCNC(N)=[NH2+])C(=O)N[C@@]([H])(CC(C)C)C([O-])=O	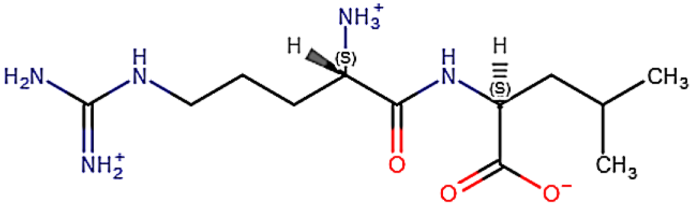

GF	7591; 8782; 9488	[H][C@@](Cc1ccccc1)(NC(=O)C[NH3+])C([O-])=O	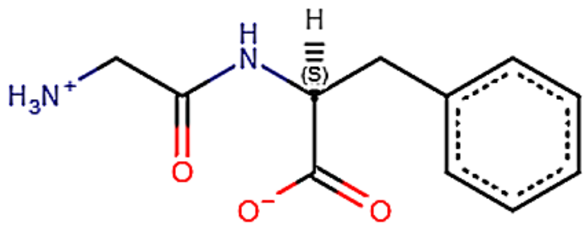
SF	7685; 8891; 9432	[H][C@]([NH3+])(CO)C(=O)N[C@@]([H])(Cc1ccccc1)C([O-])=O	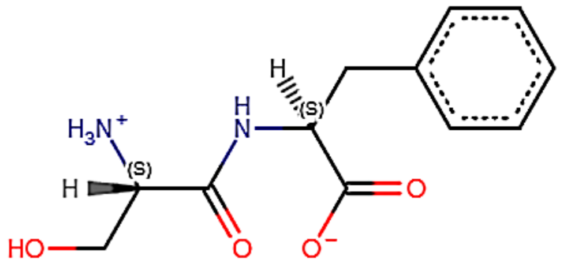
TF	8185; 8900; 9471; 9486	[H][C@](C)(O)[C@]([H])([NH3+])C(=O)N[C@@]([H])(Cc1ccccc1)C([O-])=O	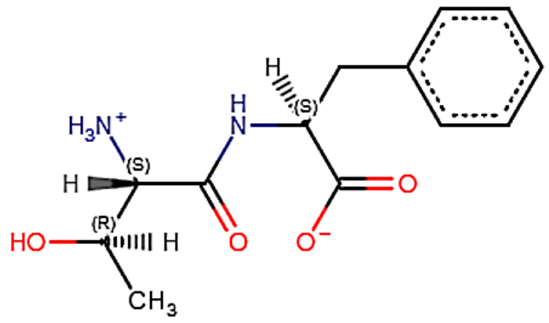
QF	8870; 9431	[H][C@]([NH3+])(CCC(N)=O)C(=O)N[C@@]([H])(Cc1ccccc1)C([O-])=O	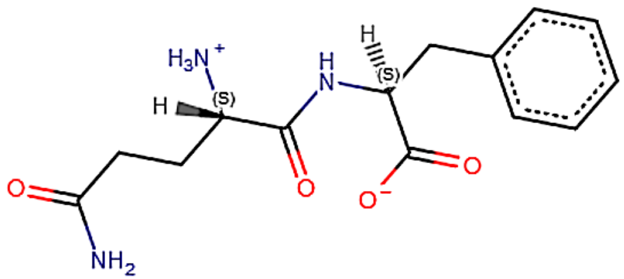

DF	9074	[H][C@]([NH3+])(CC([O-])=O)C(=O)N[C@@]([H])(Cc1ccccc1)C([O-])=O	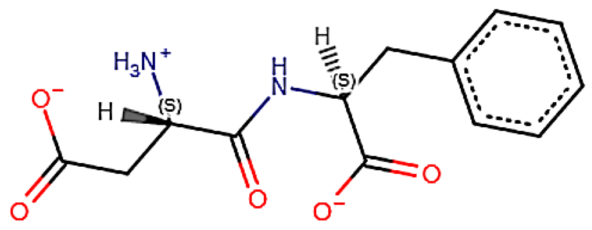
DR	8769	[H][C@]([NH3+])(CC([O-])=O)C(=O)N[C@@]([H])(CCCNC(N)=[NH2+])C([O-])=O	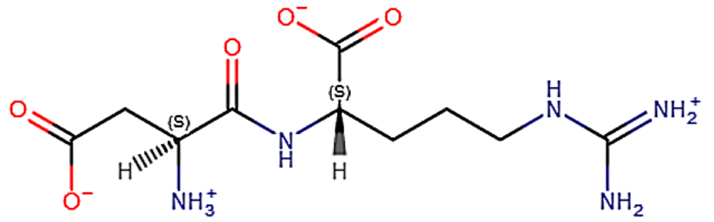
GR	7603	[H][C@@](CCCNC(N)=[NH2+])(NC(=O)C[NH3+])C([O-])=O	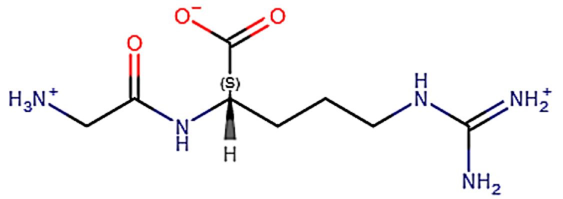

1. This column contains links to peptide data in the BIOPEP-UWM database.
2. Blue – positively charged basic groups, red – negatively charged acidic group

Table S2. Predicted targets for PGL peptide. Red font indicates 15 most likely targets.

Order:	"Target","Common name","Uniprot ID","ChEMBL ID","Target Class","Probability"
1	Dipeptidyl peptidase IV,"DPP4","P27487","CHEMBL284","Protease","0.526361274524"
2	Angiotensin-converting enzyme,"ACE","P12821","CHEMBL1808","Protease","0.444639417769"
3	Cyclooxygenase-2,"PTGS2","P35354","CHEMBL230","Oxidoreductase","0.420066875222"
4	Inhibitor of apoptosis protein 3,"XIAP","P98170","CHEMBL4198","Other cytosolic protein","0.288928252457"
5	Calpain 1,"CAPN1","P07384","CHEMBL3891","Protease","0.190656977035"
6	Sodium/glucose cotransporter 1,"SLC5A1","P13866","CHEMBL4979","Electrochemical transporter","0.17427075329"
7	HLA class I histocompatibility antigen A-3,"HLA-A","P04439","CHEMBL2632","Surface antigen","0.157929217109"
8	Leucine aminopeptidase,"LAP3","P28838","CHEMBL3965","Protease","0.133337961498",
9	Cathepsin (B and K),"CTSB","P07858","CHEMBL4072","Protease","0.125142648574"
10	Beta-secretase 1,"BACE1","P56817","CHEMBL4822","Protease","0.116965063224"
11	Neprilysin (by homology),"MME","P08473","CHEMBL1944","Protease","0.116965063224"
12	Protein farnesyltransferase,"FNTA FNTB","P49354 P49356","CHEMBL2094108","Enzyme","0.116965063224"
13	Pepsinogen C (by homology),"PGC","P20142","CHEMBL2136","Protease","0.108770969359"
14	Lipoxin A4 receptor (by homology),"FPR2","P25090","CHEMBL4227","Family A G protein-coupled receptor","0.108770969359"
15	NAD-dependent deacetylase sirtuin 2,"SIRT2","Q8IXJ6","CHEMBL4462","Eraser","0.108770969359"
16	NAD-dependent deacetylase sirtuin 1,"SIRT1","Q96EB6","CHEMBL4506","Eraser","0.108770969359"
17	Neurotensin receptor 1 (by homology),"NTSR1","P30989","CHEMBL4123","Family A G protein-coupled receptor","0.108770969359"
18	Xaa-Pro dipeptidase,"PEPD","P12955","CHEMBL4185","Protease","0.108770969359"
19	Dopamine D2 receptor,"DRD2","P14416","CHEMBL217","Family A G protein-coupled receptor","0.108770969359"
20	Baculoviral IAP repeat-containing protein 3,"BIRC3","Q13489","CHEMBL5335","Enzyme","0.100578902067"
21	Baculoviral IAP repeat-containing protein 2,"BIRC2","Q13490","CHEMBL5462","Enzyme","0.100578902067"
22	Neurotensin receptor 2,"NTSR2","O95665","CHEMBL2514","Family A G protein-coupled receptor","0.100578902067"
23	Protein kinase C epsilon,"PRKCE","Q02156","CHEMBL3582","Kinase","0.100578902067"
24	TRAIL receptor-1,"TNFRSF10A","O00220","CHEMBL3551","Membrane receptor","0.100578902067"
25	Disks large homolog 4,"DLG4","P78352","CHEMBL5666","Unclassified protein","0.100578902067"
26	Complement factor B,"CFB","P00751","CHEMBL5731","Protease","0.100578902067"
27	11-beta-hydroxysteroid dehydrogenase 1,"HSD11B1","P28845","CHEMBL4235","Enzyme","0.100578902067"
28	Furin,"FURIN","P09958","CHEMBL2611","Protease","0.100578902067"
29	Integrin alpha-IIb/beta-3,"ITGA2B ITGB3","P08514 P05106","CHEMBL2093869","Membrane receptor","0.100578902067"
30	Prolyl endopeptidase,"PREP","P48147","CHEMBL3202","Protease","0.100578902067"

31	Cathepsin D,"CTSD","P07339","CHEMBL2581","Protease","0.100578902067"
32	Aminopeptidase N,"ANPEP","P15144","CHEMBL1907","Protease","0.100578902067"
33	Matrix metalloproteinase 2,"MMP2","P08253","CHEMBL333","Protease","0.100578902067"
34	Xaa-Pro aminopeptidase 1,"XPNPEP1","Q9NQW7","CHEMBL3782","Protease","0.100578902067"
35	Xaa-Pro aminopeptidase 2,"XPNPEP2","O43895","CHEMBL4610","Protease","0.100578902067"
36	Leukotriene A4 hydrolase,"LTA4H","P09960","CHEMBL4618","Protease","0.100578902067"
37	Fibroblast activation protein alpha,"FAP","Q12884","CHEMBL4683","Protease","0.100578902067"
38	Subtilisin/kexin type 6,"PCSK6","P29122","CHEMBL2951","Protease","0.100578902067"
39	Hepatocyte growth factor activator,"HGFAC","Q04756","CHEMBL3351190","Unclassified protein","0.100578902067"
40	Histone deacetylase 3,"HDAC3","O15379","CHEMBL1829","Eraser","0.100578902067"
41	Histone deacetylase 2,"HDAC2","Q92769","CHEMBL1937","Eraser","0.100578902067"
42	Histone deacetylase 4,"HDAC4","P56524","CHEMBL3524","Eraser","0.100578902067"
43	Glyoxalase I,"GLO1","Q04760","CHEMBL2424","Enzyme","0.100578902067"
44	Plasma kallikrein,"KLKB1","P03952","CHEMBL2000","Protease","0.100578902067"
45	Integrin alpha-2/beta-3,"ITGA2 ITGB3","P17301 P05106","CHEMBL2111461","Membrane receptor","0.100578902067"
46	Integrin alpha-V/beta-3,"ITGAV ITGB3","P06756 P05106","CHEMBL1907598","Membrane receptor","0.100578902067"
47	Thrombin,"F2","P00734","CHEMBL204","Protease","0.100578902067"
48	Transcription factor AP1,"FOS JUN","P01100 P05412","CHEMBL2111421","Transcription factor","0.100578902067"
49	Beta-3 adrenergic receptor,"ADRB3","P13945","CHEMBL246","Family A G protein-coupled receptor","0.100578902067"
50	Caspase-1,"CASP1","P29466","CHEMBL4801","Protease","0.100578902067"
51	Norepinephrine transporter,"SLC6A2","P23975","CHEMBL222","Electrochemical transporter","0.100578902067"
52	Cathepsin E,"CTSE","P14091","CHEMBL3092","Protease","0.100578902067"
53	Pepsin A,"PGA5","P0DJD9","CHEMBL3295","Protease","0.100578902067"
54	ADAM9,"ADAM9","Q13443","CHEMBL5982","Protease","0.100578902067"
55	Cathepsin L,"CTSL","P07711","CHEMBL3837","Protease","0.100578902067"
56	Elastase 1,"CELA1","Q9UNI1","CHEMBL3000","Protease","0.100578902067"
57	WD repeat-containing protein 5,"WDR5","P61964","CHEMBL1075317","Unclassified protein","0.100578902067"
58	Calpain 1,"CAPN1 CAPNS1","P07384 P04632","CHEMBL2111357","Protease","0.100578902067"
59	Glycine transporter 1,"SLC6A9","P48067","CHEMBL2337","Electrochemical transporter","0.100578902067"
60	Caspase-3,"CASP3","P42574","CHEMBL2334","Protease","0.100578902067"
61	Aminopeptidase A,"ENPEP","Q07075","CHEMBL3439","Protease","0.100578902067"
62	HLA class II histocompatibility antigen DRB3-1,"HLA-DRB3","P79483","CHEMBL3460","Surface antigen","0.100578902067"
63	Probable G-protein coupled receptor 142,"GPR142","Q7Z601","CHEMBL2069161","Family A G protein-coupled receptor","0.100578902067"

64	Dipeptidyl peptidase VIII,"DPP8","Q6V1X1","CHEMBL4657","Protease","0.100578902067"
65	Dipeptidyl peptidase IX,"DPP9","Q86TI2","CHEMBL4793","Protease","0.100578902067"
66	Thyrotropin-releasing hormone receptor (by homology),"TRHR","P34981","CHEMBL1810","Family A G protein-coupled receptor","0.100578902067"
67	Renin,"REN","P00797","CHEMBL286","Protease","0.100578902067"
68	Epidermal growth factor receptor erbB1,"EGFR","P00533","CHEMBL203","Kinase","0.100578902067"
69	Casein kinase II alpha,"CSNK2A1","P68400","CHEMBL3629","Kinase","0.100578902067"
70	Serine/threonine-protein kinase PIM1,"PIM1","P11309","CHEMBL2147","Kinase","0.100578902067"
71	Serine/threonine-protein kinase PIM2,"PIM2","Q9P1W9","CHEMBL4523","Kinase","0.100578902067"
72	Dipeptidyl peptidase II,"DPP7","Q9UHL4","CHEMBL3976","Protease","0.100578902067"
73	Tyrosyl-tRNA synthetase,"YARS","P54577","CHEMBL3179","Enzyme","0.100578902067"
74	Mu opioid receptor,"OPRM1","P35372","CHEMBL233","Family A G protein-coupled receptor","0.100578902067"
75	Delta opioid receptor,"OPRD1","P41143","CHEMBL236","Family A G protein-coupled receptor","0.100578902067"
76	Lysine-specific demethylase 4A,"KDM4A","O75164","CHEMBL5896","Eraser","0.100578902067"
77	Lysine-specific demethylase 4C,"KDM4C","Q9H3R0","CHEMBL6175","Eraser","0.100578902067"
78	Lysine-specific demethylase 5C,"KDM5C","P41229","CHEMBL2163176","Eraser","0.100578902067"
79	Lysine-specific demethylase 5B,"KDM5B","Q9UGL1","CHEMBL3774295","Eraser","0.100578902067"
80	Lysine-specific demethylase 2B,"KDM2B","Q8NHM5","CHEMBL3779760","Eraser","0.100578902067"
81	Histone deacetylase 1,"HDAC1","Q13547","CHEMBL325","Eraser","0.100578902067"
82	Histamine H1 receptor,"HRH1","P35367","CHEMBL231","Family A G protein-coupled receptor","0.100578902067"
83	Caspase-8,"CASP8","Q14790","CHEMBL3776","Protease","0.100578902067"
84	MAP kinase signal-integrating kinase 2,"MKNK2","Q9HBH9","CHEMBL4204","Kinase","0.100578902067"
85	Leukocyte elastase,"ELANE","P08246","CHEMBL248","Protease","0.100578902067"
86	Thrombin and coagulation factor X,"F10","P00742","CHEMBL244","Protease","0.100578902067"
87	Lysine-specific demethylase 5A,"KDM5A","P29375","CHEMBL2424504","Eraser","0.100578902067"
88	Squalene synthetase (by homology),"FDFT1","P37268","CHEMBL3338","Enzyme","0.100578902067"
89	Trypsin I,"PRSS1","P07477","CHEMBL209","Protease","0.100578902067"
90	Serine/threonine-protein kinase Aurora-A,"AURKA","O14965","CHEMBL4722","Kinase","0.100578902067"
91	Poly [ADP-ribose] polymerase-1,"PARP1","P09874","CHEMBL3105","Enzyme","0.100578902067"
92	Glutathione S-transferase kappa 1,"GSTK1","Q9Y2Q3","CHEMBL4491","Enzyme","0.100578902067"
93	Glycogen synthase kinase-3 beta,"GSK3B","P49841","CHEMBL262","Kinase","0.100578902067"
94	Calcium sensing receptor,"CASR","P41180","CHEMBL1878","Family C G protein-coupled receptor","0.100578902067"
95	GABA transporter 1 (by homology),"SLC6A1","P30531","CHEMBL1903","Electrochemical transporter","0.100578902067"
96	Endoplasmic reticulum aminopeptidase 2,"ERAP2","Q6P179","CHEMBL5043","Protease","0.100578902067"

97	Carboxypeptidase B,"CPB1","P15086","CHEMBL2552","Protease","0.100578902067"
98	AMP deaminase 3,"AMPD3","Q01432","CHEMBL2912","Enzyme","0.100578902067"
99	Ribosomal protein S6 kinase 1,"RPS6KB1","P23443","CHEMBL4501","Kinase","0.100578902067"
100	Proteasome Macropain subunit,"PSMB2","P49721","CHEMBL3492","Protease","0.100578902067"

Table S3. Predicted targets for RL peptide. Red font indicates 15 most likely targets.

Order:	"Target","Common name","Uniprot ID","ChEMBL ID","Target Class","Probability"
1	Neurotensin receptor 2,"NTSR2","O95665","CHEMBL2514","Family A G protein-coupled receptor","0.166097445484"
2	Complement factor B,"CFB","P00751","CHEMBL5731","Protease","0.166097445484"
3	Subtilisin/kexin type 6,"PCSK6","P29122","CHEMBL2951","Protease","0.133337961498"
4	Hepatocyte growth factor activator,"HGFAC","Q04756","CHEMBL3351190","Unclassified protein"
5	Thrombin and coagulation factor X,"F10","P00742","CHEMBL244","Protease"
6	WD repeat-containing protein 5,"WDR5","P61964","CHEMBL1075317","Unclassified protein"
7	Furin,"FURIN","P09958","CHEMBL2611","Protease","0.108770969359"
8	Neurotensin receptor 1,"NTSR1","P30989","CHEMBL4123","Family A G protein-coupled receptor","0.100578902067"
9	Epoxide hydratase (by homology),"EPHX2","P34913","CHEMBL2409","Protease","0.100578902067"
10	Integrin alpha-V/beta-3,"ITGAV ITGB3","P06756 P05106","CHEMBL1907598","Membrane receptor","0.100578902067"
11	Neuropilin-1 (by homology),"NRP1","O14786","CHEMBL5174","Secreted protein","0.100578902067"
12	Integrin alpha-IIb/beta-3,"ITGA2B ITGB3","P08514 P05106","CHEMBL2093869","Membrane receptor","0.100578902067"
13	Proteinase-activated receptor 2,"F2RL1","P55085","CHEMBL5963","Family A G protein-coupled receptor","0.100578902067"
14	c-Jun N-terminal kinase 1,"MAPK8","P45983","CHEMBL2276","Kinase","0.100578902067"
15	Thrombin,"F2","P00734","CHEMBL204","Protease","0.100578902067"
16	Integrin alpha-5/beta-1,"ITGB1 ITGA5","P05556 P08648","CHEMBL2095226","Membrane receptor","0.100578902067"
17	Transmembrane protease serine 6,"TMPRSS6","Q8IU80","CHEMBL1795139","Protease","0.100578902067"
18	Serine protease hepsin,"HPN","P05981","CHEMBL2079849","Protease","0.100578902067"
19	Dipeptidyl peptidase IV,"DPP4","P27487","CHEMBL284","Protease","0.100578902067"
20	Angiotensin-converting enzyme,"ACE","P12821","CHEMBL1808","Protease","0.100578902067"
21	HLA class I histocompatibility antigen A-3,"HLA-A","P04439","CHEMBL2632","Surface antigen","0.100578902067"
22	Integrin alpha-V/beta-5,"ITGB5 ITGAV","P18084 P06756","CHEMBL2096675","Membrane receptor","0.100578902067"
23	Nitric oxide synthase, inducible,"NOS2","P35228","CHEMBL4481","Enzyme","0.100578902067"
24	Plasminogen,"PLG","P00747","CHEMBL1801","Protease","0.100578902067"
25	Cathepsin (H and K),"CTSH","P09668","CHEMBL2225","Protease","0.100578902067"
26	Cathepsin L,"CTSL","P07711","CHEMBL3837","Protease","0.100578902067"
27	Calpain 1,"CAPN1","P07384","CHEMBL3891","Protease","0.100578902067"
28	Cathepsin (B and K),"CTSB","P07858","CHEMBL4072","Protease","0.100578902067"
29	Inhibitor of apoptosis protein 3,"XIAP","P98170","CHEMBL4198","Other cytosolic protein","0.100578902067"
30	Menin,"MEN1","O00255","CHEMBL1615381","Unclassified protein","0.100578902067"

31	Pepsinogen C (by homology),"PGC","P20142","CHEMBL2136","Protease","0.100578902067"
32	Matriptase,"ST14","Q9Y5Y6","CHEMBL3018","Protease","0.100578902067","2 / 3Â Â Â Â Â "
33	Cyclin-dependent kinase 2/cyclin A,"CDK2 CCNA1 CCNA2","P24941 P78396 P20248","CHEMBL2094128","Other cytosolic protein","0.100578902067"
34	Tyrosyl-tRNA synthetase,"YARS","P54577","CHEMBL3179","Enzyme","0.100578902067"
35	Hydroxycarboxylic acid receptor 2,"HCAR2","Q8TDS4","CHEMBL3785","Family A G protein-coupled receptor","0.100578902067"
36	Carboxypeptidase B2 isoform A,"CPB2","Q96IY4","CHEMBL3419","Protease","0.100578902067"
37	Folylpoly-gamma-glutamate synthetase,"FPGS","Q05932","CHEMBL3171","Enzyme","0.100578902067"
38	Neprilysin,"MME","P08473","CHEMBL1944","Protease","0.100578902067"
39	Cyclin-dependent kinase 4/cyclin D1,"CCND1 CDK4","P24385 P11802","CHEMBL1907601","Kinase","0.100578902067"
40	CDK2/Cyclin A,"CCNA2 CDK2","P20248 P24941","CHEMBL3038469","Kinase","0.100578902067"
41	Dipeptidyl peptidase VIII,"DPP8","Q6V1X1","CHEMBL4657","Protease","0.100578902067"
42	Muscarinic acetylcholine receptor M4,"CHRM4","P08173","CHEMBL1821","Family A G protein-coupled receptor","0.100578902067"
43	Muscarinic acetylcholine receptor M2,"CHRM2","P08172","CHEMBL211","Family A G protein-coupled receptor","0.100578902067"
44	Muscarinic acetylcholine receptor M1,"CHRM1","P11229","CHEMBL216","Family A G protein-coupled receptor","0.100578902067"
45	Integrin alpha-2/beta-3,"ITGA2 ITGB3","P17301 P05106","CHEMBL2111461","Membrane receptor","0.100578902067"
46	Endoplasmic reticulum aminopeptidase 2,"ERAP2","Q6P179","CHEMBL5043","Protease","0.100578902067"

Table S4. Predicted targets for GF peptide. Red font indicates 15 most likely targets.

Order:	"Target","Common name","Uniprot ID","ChEMBL ID","Target Class","Probability"
1	Oligopeptide transporter small intestine isoform,"SLC15A1","P46059","CHEMBL4605","Electrochemical transporter","0.129815190994"
2	Calpain 1,"CAPN1","P07384","CHEMBL3891","Protease","0.112450964818"
3	Neprilysin (by homology),"MME","P08473","CHEMBL1944","Protease","0.103761755413"
4	Cyclooxygenase-2,"PTGS2","P35354","CHEMBL230","Oxidoreductase","0.0777583259988"
5	Tyrosyl-tRNA synthetase,"YARS","P54577","CHEMBL3179","Enzyme","0.0690974435253"
6	Inhibitor of apoptosis protein 3,"XIAP","P98170","CHEMBL4198","Other cytosolic protein","0.0690974435253"
7	HLA class I histocompatibility antigen A-3,"HLA-A","P04439","CHEMBL2632","Surface antigen","0.0604245879294"
8	Angiotensin-converting enzyme,"ACE","P12821","CHEMBL1808","Protease","0.0604245879294"
9	Solute carrier family 22 member 6 (by homology),"SLC22A6","Q4U2R8","CHEMBL1641347","Electrochemical transporter","0.0604245879294"
10	Cholecystokinin B receptor,"CCKBR","P32239","CHEMBL298","Family A G protein-coupled receptor","0.0604245879294"
11	Neurokinin 1 receptor,"TACR1","P25103","CHEMBL249","Family A G protein-coupled receptor","0.0604245879294"
12	Carboxypeptidase A1,"CPA1","P15085","CHEMBL2088","Protease","0.0604245879294"
13	Angiotensin-converting enzyme 2,"ACE2","Q9BYF1","CHEMBL3736","Protease","0.0604245879294"
14	Carboxypeptidase B,"CPB1","P15086","CHEMBL2552","Protease","0.0604245879294"
15	Proenkephalin B,"PDYN","P01213","CHEMBL2227","Other ion channel","0.0604245879294"
16	Formyl peptide receptor 1,"FPR1","P21462","CHEMBL3359","Family A G protein-coupled receptor","0.0604245879294"
17	Lysine-specific demethylase 4C,"KDM4C","Q9H3R0","CHEMBL6175","Eraser","0.0604245879294"
18	Ephrin type-A receptor 2,"EPHA2","P29317","CHEMBL2068","Kinase","0.0604245879294"
19	Cathepsin K,"CTSK","P43235","CHEMBL268","Protease","0.0604245879294"
20	Cathepsin (B and K),"CTSB","P07858","CHEMBL4072","Protease","0.0604245879294"
21	Chromobox protein homolog 7,"CBX7","O95931","CHEMBL1764946","Reader","0.0604245879294"
22	E3 SUMO-protein ligase CBX4,"CBX4","O00257","CHEMBL3232685","Enzyme","0.0604245879294"
23	Serotonin 2b (5-HT2b) receptor,"HTR2B","P41595","CHEMBL1833","Family A G protein-coupled receptor","0.0604245879294"
24	Nitric oxide synthase, inducible,"NOS2","P35228","CHEMBL4481","Enzyme","0.0604245879294"
25	Aminopeptidase N,"ANPEP","P15144","CHEMBL1907","Protease","0.0604245879294"
26	Glutamate receptor ionotropic, AMPA 1,"GRIA1","P42261","CHEMBL2009","Ligand-gated ion channel","0.0604245879294"
27	Dipeptidyl peptidase I,"CTSC","P53634","CHEMBL2252","Protease","0.0604245879294"
28	Epoxide hydratase,"EPHX2","P34913","CHEMBL2409","Protease","0.0604245879294"
29	Kynureninase,"KYNU","Q16719","CHEMBL5100","Enzyme","0.0604245879294"

30	Voltage-gated calcium channel alpha2/delta subunit 1 (by homology),"CACNA2D1","P54289","CHEMBL1919","Calcium channel auxiliary subunit alpha2delta family","0.0604245879294"
31	Nitric-oxide synthase, endothelial,"NOS3","P29474","CHEMBL4803","Enzyme","0.0604245879294"
32	Cathepsin L,"CTSL","P07711","CHEMBL3837","Protease","0.0604245879294",
33	Cyclin-dependent kinase 4/cyclin D1,"CCND1 CDK4","P24385 P11802","CHEMBL1907601","Kinase","0.0604245879294"
34	CDK2/Cyclin A,"CCNA2 CDK2","P20248 P24941","CHEMBL3038469","Kinase","0.0604245879294"
35	Intercellular adhesion molecule (ICAM-1), Integrin alpha-L/beta-2,"ITGAL ICAM1 ITGB2","P20701 P05362 P05107","CHEMBL2096661","Membrane receptor","0.0604245879294"
36	Leukotriene A4 hydrolase,"LTA4H","P09960","CHEMBL4618","Protease","0.0604245879294"
37	MAP kinase-activated protein kinase 2,"MAPKAPK2","P49137","CHEMBL2208","Kinase","0.0604245879294"
38	Mu opioid receptor (by homology),"OPRM1","P35372","CHEMBL233","Family A G protein-coupled receptor","0.0604245879294"
39	MAP kinase ERK2,"MAPK1","P28482","CHEMBL4040","Kinase","0.0604245879294"
40	Neurotensin receptor 1 (by homology),"NTSR1","P30989","CHEMBL4123","Family A G protein-coupled receptor","0.0604245879294"
41	Caspase-1,"CASP1","P29466","CHEMBL4801","Protease","0.0604245879294"
42	GABA-B receptor (by homology),"GABBR1","Q9UBS5","CHEMBL2064","Family C G protein-coupled receptor","0.0604245879294"
43	Neurotensin receptor 2,"NTSR2","O95665","CHEMBL2514","Family A G protein-coupled receptor","0.0604245879294"
44	Integrin alpha-4/beta-1,"ITGB1 ITGA4","P05556 P13612","CHEMBL1907599","Membrane receptor","0.0604245879294"
45	Cyclin-dependent kinase 2/cyclin A,"CDK2 CCNA1 CCNA2","P24941 P78396 P20248","CHEMBL2094128","Other cytosolic protein","0.0604245879294"
46	C-C chemokine receptor type 3,"CCR3","P51677","CHEMBL3473","Family A G protein-coupled receptor","0.0604245879294"
47	Sigma opioid receptor,"SIGMAR1","Q99720","CHEMBL287","Membrane receptor","0.0604245879294"
48	Delta opioid receptor (by homology),"OPRD1","P41143","CHEMBL236","Family A G protein-coupled receptor","0.0604245879294"
49	HLA class II histocompatibility antigen DRB1-1,"HLA-DRB1","P04229","CHEMBL1943","Surface antigen","0.0604245879294"
50	Ribonucleoside-diphosphate reductase M1 chain (by homology),"RRM1","P23921","CHEMBL1830","Oxidoreductase","0.0604245879294"
51	Gamma-secretase,"PSENEN","Q9NZ42","CHEMBL2374","Enzyme","0.0604245879294"
52	Peptidyl-glycine alpha-amidating monooxygenase,"PAM","P19021","CHEMBL2544","Enzyme","0.0604245879294"
53	Dipeptidyl peptidase IV,"DPP4","P27487","CHEMBL284","Protease","0.0604245879294"
54	Serine/threonine-protein kinase PIM1,"PIM1","P11309","CHEMBL2147","Kinase","0.0604245879294"
55	Serine/threonine-protein kinase PIM2,"PIM2","Q9P1W9","CHEMBL4523","Kinase","0.0604245879294"
56	Glutamate receptor ionotropic, AMPA 2,"GRIA2","P42262","CHEMBL4016","Ligand-gated ion channel","0.0604245879294"
57	Neurokinin 2 receptor,"TACR2","P21452","CHEMBL2327","Family A G protein-coupled receptor","0.0604245879294"
58	Acyl-CoA:dihydroxyacetonephosphateacyltransferase,"GNPAT","O15228","CHEMBL4494","Enzyme","0.0604245879294"
59	Lysine-specific demethylase 5C,"KDM5C","P41229","CHEMBL2163176","Eraser","0.0604245879294"
60	Lysine-specific demethylase 4B,"KDM4B","O94953","CHEMBL3313832","Eraser","0.0604245879294"

61	Lysine-specific demethylase 5B,"KDM5B","Q9UGL1","CHEMBL3774295","Eraser","0.0604245879294"
62	Lysine-specific demethylase 4A,"KDM4A","O75164","CHEMBL5896","Eraser","0.0604245879294"
63	Metabotropic glutamate receptor 6 (by homology),"GRM6","O15303","CHEMBL4573","Family C G protein-coupled receptor","0.0604245879294"
64	Metabotropic glutamate receptor 2,"GRM2","Q14416","CHEMBL5137","Family C G protein-coupled receptor","0.0604245879294"
65	Galanin receptor 1 (by homology),"GALR1","P47211","CHEMBL4894","Family A G protein-coupled receptor","0.0604245879294"
66	Galanin receptor 2 (by homology),"GALR2","O43603","CHEMBL3176","Family A G protein-coupled receptor","0.0604245879294"

Table S5. Predicted targets for SF peptide. Red font indicates 15 most likely targets.

Order:	"Target","Common name","Uniprot ID","ChEMBL ID","Target Class","Probability"
1	Calpain 1,"CAPN1","P07384","CHEMBL3891","Protease","0.0807923867549"
2	Oligopeptide transporter small intestine isoform,"SLC15A1","P46059","CHEMBL4605","Electrochemical transporter","0.071715932485"
3	Cyclooxygenase-2,"PTGS2","P35354","CHEMBL230","Oxidoreductase","0.0626219668353"
4	Angiotensin-converting enzyme,"ACE","P12821","CHEMBL1808","Protease","0.0626219668353"
5	Neprilysin,"MME","P08473","CHEMBL1944","Protease","0.0626219668353"
6	Tyrosyl-tRNA synthetase,"YARS","P54577","CHEMBL3179","Enzyme","0.0626219668353"
7	Inhibitor of apoptosis protein 3,"XIAP","P98170","CHEMBL4198","Other cytosolic protein","0.0535560755162"
8	HLA class I histocompatibility antigen A-3,"HLA-A","P04439","CHEMBL2632","Surface antigen","0.0535560755162"
9	Mu opioid receptor (by homology),"OPRM1","P35372","CHEMBL233","Family A G protein-coupled receptor","0.0535560755162"
10	Solute carrier family 22 member 6 (by homology),"SLC22A6","Q4U2R8","CHEMBL1641347","Electrochemical transporter","0.0535560755162"
11	Chromobox protein homolog 7,"CBX7","O95931","CHEMBL1764946","Reader","0.0535560755162"
12	E3 SUMO-protein ligase CBX4,"CBX4","O00257","CHEMBL3232685","Enzyme","0.0535560755162"
13	Ribonucleoside-diphosphate reductase M1 chain (by homology),"RRM1","P23921","CHEMBL1830","Oxidoreductase","0.0535560755162"
14	Neurokinin 2 receptor,"TACR2","P21452","CHEMBL2327","Family A G protein-coupled receptor","0.0535560755162"
15	Proenkephalin B,"PDYN","P01213","CHEMBL2227","Other ion channel","0.0535560755162"
16	Beta-secretase 1,"BACE1","P56817","CHEMBL4822","Protease","0.0535560755162"
17	Formyl peptide receptor 1,"FPR1","P21462","CHEMBL3359","Family A G protein-coupled receptor","0.0535560755162"
18	Calcitonin gene-related peptide type 1 receptor,"CALCRL","Q16602","CHEMBL3798","Family B G protein-coupled receptor","0.0535560755162"
19	Ephrin type-A receptor 2,"EPHA2","P29317","CHEMBL2068","Kinase","0.0535560755162"
20	Cholecystokinin B receptor,"CCKBR","P32239","CHEMBL298","Family A G protein-coupled receptor","0.0535560755162"
21	Neurokinin 1 receptor (by homology),"TACR1","P25103","CHEMBL249","Family A G protein-coupled receptor","0.0535560755162"
22	Bradykinin B1 receptor,"BDKRB1","P46663","CHEMBL4308","Family A G protein-coupled receptor","0.0535560755162"
23	Neuropeptide FF receptor 1,"NPFFR1","Q9GZQ6","CHEMBL5951","Family A G protein-coupled receptor","0.0535560755162"
24	Neuropeptide FF receptor 2,"NPFFR2","Q9Y5X5","CHEMBL5952","Family A G protein-coupled receptor","0.0535560755162"
25	Delta opioid receptor (by homology),"OPRD1","P41143","CHEMBL236","Family A G protein-coupled receptor","0.0535560755162"
26	Endothelin receptor ET-A,"EDNRA","P25101","CHEMBL252","Family A G protein-coupled receptor","0.0535560755162"
27	Carboxypeptidase A1,"CPA1","P15085","CHEMBL2088","Protease","0.0535560755162"
28	Epoxide hydratase,"EPHX2","P34913","CHEMBL2409","Protease","0.0535560755162"
29	Galanin receptor 1 (by homology),"GALR1","P47211","CHEMBL4894","Family A G protein-coupled receptor","0.0535560755162"
30	Galanin receptor 2 (by homology),"GALR2","O43603","CHEMBL3176","Family A G protein-coupled receptor","0.0535560755162"

31	Cathepsin D,"CTSD","P07339","CHEMBL2581","Protease","0.0535560755162"
32	Cathepsin E,"CTSE","P14091","CHEMBL3092","Protease","0.0535560755162"
33	Carboxypeptidase B,"CPB1","P15086","CHEMBL2552","Protease","0.0535560755162"
34	Sphingosine kinase 1,"SPHK1","Q9NYA1","CHEMBL4394","Enzyme","0.0535560755162"
35	Lysine-specific demethylase 4C,"KDM4C","Q9H3R0","CHEMBL6175","Eraser","0.0535560755162"
36	Cyclin-dependent kinase 4/cyclin D1,"CCND1 CDK4","P24385 P11802","CHEMBL1907601","Kinase","0.0535560755162"
37	CDK2/Cyclin A,"CCNA2 CDK2","P20248 P24941","CHEMBL3038469","Kinase","0.0535560755162"
38	MAP kinase ERK2,"MAPK1","P28482","CHEMBL4040","Kinase","0.0535560755162"
39	Neurotensin receptor 1 (by homology),"NTSR1","P30989","CHEMBL4123","Family A G protein-coupled receptor","0.0535560755162"
40	Glucosamine--fructose-6-phosphate aminotransferase [isomerizing] 1,"GFPT1","Q06210","CHEMBL1909481","Enzyme","0.0535560755162"
41	Signal transducer and activator of transcription 3,"STAT3","P40763","CHEMBL4026","Transcription factor","0.0535560755162"
42	Cathepsin (B and K),"CTSB","P07858","CHEMBL4072","Protease","0.0535560755162"
43	Ghrelin receptor,"GHSR","Q92847","CHEMBL4616","Family A G protein-coupled receptor","0.0535560755162"
44	Serine/threonine-protein kinase/endoribonuclease IRE1,"ERN1","O75460","CHEMBL1163101","Enzyme","0.0535560755162"
45	Pyroglutamylated RFamide peptide receptor,"QRFPR","Q96P65","CHEMBL5852","Family A G protein-coupled receptor","0.0535560755162"
46	Beta secretase 2,"BACE2","Q9Y5Z0","CHEMBL2525","Protease","0.0535560755162"
47	Nitric oxide synthase, inducible,"NOS2","P35228","CHEMBL4481","Enzyme","0.0535560755162"
48	Interleukin-1 beta,"IL1B","P01584","CHEMBL1909490","Secreted protein","0.0535560755162"
49	Cyclin-dependent kinase 2/cyclin A,"CDK2 CCNA1 CCNA2","P24941 P78396 P20248","CHEMBL2094128","Other cytosolic protein","0.0535560755162
50	C-C chemokine receptor type 3,"CCR3","P51677","CHEMBL3473","Family A G protein-coupled receptor","0.0535560755162"
51	Kappa Opioid receptor,"OPRK1","P41145","CHEMBL237","Family A G protein-coupled receptor","0.0535560755162"
52	Neurotensin receptor 2,"NTSR2","O95665","CHEMBL2514","Family A G protein-coupled receptor","0.0535560755162"
53	Cathepsin L,"CTSL","P07711","CHEMBL3837","Protease","0.0535560755162"
54	Integrin alpha-V/beta-3,"ITGAV ITGB3","P06756 P05106","CHEMBL1907598","Membrane receptor","0.0535560755162"
55	Gamma-secretase,"PSENEN","Q9NZ42","CHEMBL2374","Enzyme","0.0535560755162"
56	Vasopressin V1a receptor,"AVPR1A","P37288","CHEMBL1889","Family A G protein-coupled receptor","0.0535560755162"
57	Nitric-oxide synthase, endothelial,"NOS3","P29474","CHEMBL4803","Enzyme","0.0535560755162"
58	Kynureninase,"KYNU","Q16719","CHEMBL5100","Enzyme","0.0535560755162"
59	C-X-C chemokine receptor type 7,"ACKR3","P25106","CHEMBL2010631","Family A G protein-coupled receptor","0.0535560755162"
60	C3a anaphylatoxin chemotactic receptor,"C3AR1","Q16581","CHEMBL4761","Family A G protein-coupled receptor","0.0535560755162"
61	Glutamate receptor ionotropic kainate 2,"GRIK2","Q13002","CHEMBL3683","Ligand-gated ion channel","0.0535560755162"
62	Glutamate receptor ionotropic kainate 3,"GRIK3","Q13003","CHEMBL3684","Ligand-gated ion channel","0.0535560755162"
63	Protein farnesyltransferase,"FNTA FNTB","P49354 P49356","CHEMBL2094108","Enzyme","0.0535560755162"

64	Cathepsin K,"CTSK","P43235","CHEMBL268","Protease","0.0535560755162"
65	Kynurenine 3-monooxygenase (by homology),"KMO","O15229","CHEMBL2145","Oxidoreductase","0.0535560755162"
66	MAP kinase-activated protein kinase 2,"MAPKAPK2","P49137","CHEMBL2208","Kinase","0.0535560755162"
67	Geranylgeranyl transferase type I,"PGGT1B FNTA","P53609 P49354","CHEMBL2095164","Enzyme","0.0535560755162"
68	Renin,"REN","P00797","CHEMBL286","Protease","0.0535560755162"
69	Metabotropic glutamate receptor 2 (by homology),"GRM2","Q14416","CHEMBL5137","Family C G protein-coupled receptor","0.0535560755162"

Table S6. Predicted targets for TF peptide. Red font indicates 15 most likely targets.

Order:	"Target","Common name","Uniprot ID","ChEMBL ID","Target Class","Probability"
1	Calpain 1,"CAPN1","P07384","CHEMBL3891","Protease","0.237759864997"
2	Tyrosyl-tRNA synthetase,"YARS","P54577","CHEMBL3179","Enzyme","0.143473412261"
3	Cyclooxygenase-2,"PTGS2","P35354","CHEMBL230","Oxidoreductase","0.143473412261"
4	Neprilysin (by homology),"MME","P08473","CHEMBL1944","Protease","0.135616202575"
5	Oligopeptide transporter small intestine isoform,"SLC15A1","P46059","CHEMBL4605","Electrochemical transporter","0.135616202575"
6	Angiotensin-converting enzyme,"ACE","P12821","CHEMBL1808","Protease","0.127750341333"
7	Mu opioid receptor (by homology),"OPRM1","P35372","CHEMBL233","Family A G protein-coupled receptor","0.119895126898"
8	HLA class I histocompatibility antigen A-3,"HLA-A","P04439","CHEMBL2632","Surface antigen","0.119895126898"
9	Solute carrier family 22 member 6 (by homology),"SLC22A6","Q4U2R8","CHEMBL1641347","Electrochemical transporter","0.112041901328"
10	Delta opioid receptor (by homology),"OPRD1","P41143","CHEMBL236","Family A G protein-coupled receptor","0.112041901328"
11	Chromobox protein homolog 7,"CBX7","O95931","CHEMBL1764946","Reader","0.112041901328"
12	E3 SUMO-protein ligase CBX4,"CBX4","O00257","CHEMBL3232685","Enzyme","0.112041901328"
13	Ribonucleoside-diphosphate reductase M1 chain (by homology),"RRM1","P23921","CHEMBL1830","Oxidoreductase","0.112041901328"
14	Neurokinin 2 receptor,"TACR2","P21452","CHEMBL2327","Family A G protein-coupled receptor","0.112041901328"
15	Proenkephalin B,"PDYN","P01213","CHEMBL2227","Other ion channel","0.112041901328"
16	Protein farnesyltransferase,"FNTA FNTB","P49354 P49356","CHEMBL2094108","Enzyme","0.112041901328"
17	Formyl peptide receptor 1,"FPR1","P21462","CHEMBL3359","Family A G protein-coupled receptor","0.112041901328"
18	Ephrin type-A receptor 2,"EPHA2","P29317","CHEMBL2068","Kinase","0.112041901328"
19	Calcitonin gene-related peptide type 1 receptor,"CALCRL","Q16602","CHEMBL3798","Family B G protein-coupled receptor","0.112041901328"
20	Cholecystokinin B receptor,"CCKBR","P32239","CHEMBL298","Family A G protein-coupled receptor","0.112041901328"
21	Neurokinin 1 receptor,"TACR1","P25103","CHEMBL249","Family A G protein-coupled receptor","0.112041901328"
22	Renin,"REN","P00797","CHEMBL286","Protease","0.112041901328"
23	Bradykinin B1 receptor,"BDKRB1","P46663","CHEMBL4308","Family A G protein-coupled receptor" ,"0.112041901328"
24	Kappa Opioid receptor,"OPRK1","P41145","CHEMBL237","Family A G protein-coupled receptor","0.112041901328"
25	Carboxypeptidase A1,"CPA1","P15085","CHEMBL2088","Protease","0.112041901328"
26	Neuropeptide FF receptor 1,"NPFFR1","Q9GZQ6","CHEMBL5951","Family A G protein-coupled receptor","0.112041901328"
27	Neuropeptide FF receptor 2,"NPFFR2","Q9Y5X5","CHEMBL5952","Family A G protein-coupled receptor","0.112041901328"
28	Galanin receptor 1 (by homology),"GALR1","P47211","CHEMBL4894","Family A G protein-coupled receptor","0.112041901328"
29	Galanin receptor 2 (by homology),"GALR2","O43603","CHEMBL3176","Family A G protein-coupled receptor","0.112041901328"
30	Epoxide hydratase,"EPHX2","P34913","CHEMBL2409","Protease","0.112041901328"

31	Integrin alpha-V/beta-3,"ITGAV ITGB3","P06756 P05106","CHEMBL1907598","Membrane receptor","0.112041901328"
32	Carboxypeptidase B,"CPB1","P15086","CHEMBL2552","Protease","0.112041901328"
33	Nitric oxide synthase, inducible,"NOS2","P35228","CHEMBL4481","Enzyme","0.112041901328"
34	Sphingosine kinase 1,"SPHK1","Q9NYA1","CHEMBL4394","Enzyme","0.112041901328"
35	Cathepsin D,"CTSD","P07339","CHEMBL2581","Protease","0.112041901328"
36	Cathepsin E,"CTSE","P14091","CHEMBL3092","Protease","0.112041901328"
37	Inhibitor of apoptosis protein 3,"XIAP","P98170","CHEMBL4198","Other cytosolic protein","0.112041901328"
38	Cathepsin (B and K),"CTSB","P07858","CHEMBL4072","Protease","0.112041901328"
39	Cyclin-dependent kinase 4/cyclin D1,"CCND1 CDK4","P24385 P11802","CHEMBL1907601","Kinase","0.112041901328"
40	CDK2/Cyclin A,"CCNA2 CDK2","P20248 P24941","CHEMBL3038469","Kinase","0.112041901328"
41	Neurotensin receptor 1 (by homology),"NTSR1","P30989","CHEMBL4123","Family A G protein-coupled receptor","0.112041901328"
42	Endothelin-converting enzyme 1,"ECE1","P42892","CHEMBL4791","Protease","0.112041901328"
43	Signal transducer and activator of transcription 3,"STAT3","P40763","CHEMBL4026","Transcription factor","0.112041901328"
44	Leukotriene A4 hydrolase,"LTA4H","P09960","CHEMBL4618","Protease","0.112041901328"
45	Caspase-1,"CASP1","P29466","CHEMBL4801","Protease","0.112041901328"
46	Aminopeptidase N,"ANPEP","P15144","CHEMBL1907","Protease","0.112041901328"
47	Dipeptidyl peptidase IV,"DPP4","P27487","CHEMBL284","Protease","0.112041901328"
48	Pyroglutamylated RFamide peptide receptor,"QRFPR","Q96P65","CHEMBL5852","Family A G protein-coupled receptor","0.112041901328"
49	HMG-CoA reductase,"HMGCR","P04035","CHEMBL402","Oxidoreductase","0.112041901328"
50	Beta secretase 2,"BACE2","Q9Y5Z0","CHEMBL2525","Protease","0.112041901328"
51	Interleukin-1 beta,"IL1B","P01584","CHEMBL1909490","Secreted protein","0.112041901328"
52	Cathepsin L,"CTSL","P07711","CHEMBL3837","Protease","0.112041901328"
53	Cyclin-dependent kinase 2/cyclin A,"CDK2 CCNA1 CCNA2","P24941 P78396 P20248","CHEMBL2094128","Other cytosolic protein","0.112041901328
54	Disks large homolog 4,"DLG4","P78352","CHEMBL5666","Unclassified protein","0.112041901328"
55	Dopamine transporter (by homology),"SLC6A3","Q01959","CHEMBL238","Electrochemical transporter","0.112041901328"
56	Neurotensin receptor 2,"NTSR2","O95665","CHEMBL2514","Family A G protein-coupled receptor","0.112041901328"
57	Vasopressin V1a receptor,"AVPR1A","P37288","CHEMBL1889","Family A G protein-coupled receptor","0.112041901328"
58	MAP kinase-activated protein kinase 2,"MAPKAPK2","P49137","CHEMBL2208","Kinase","0.112041901328"
59	Leucine aminopeptidase,"LAP3","P28838","CHEMBL3965","Protease","0.112041901328"
60	Cathepsin K,"CTSK","P43235","CHEMBL268","Protease","0.112041901328"
61	C-X-C chemokine receptor type 7,"ACKR3","P25106","CHEMBL2010631","Family A G protein-coupled receptor","0.112041901328"
62	Dipeptidyl peptidase I,"CTSC","P53634","CHEMBL2252","Protease","0.112041901328","0 / 207Â Â Â Â Â "
63	C3a anaphylatoxin chemotactic receptor,"C3AR1","Q16581","CHEMBL4761","Family A G protein-coupled receptor","0.112041901328","0 / 11Â Â Â Â Â "

64	Max-like protein X,"MLX","Q9UH92","CHEMBL2062357","Unclassified protein","0.112041901328"
65	HLA class II histocompatibility antigen DRB1-1,"HLA-DRB1","P04229","CHEMBL1943","Surface antigen","0.112041901328"
66	Nitric-oxide synthase, endothelial,"NOS3","P29474","CHEMBL4803","Enzyme","0.112041901328"
67	Serine/threonine-protein kinase/endoribonuclease IRE1,"ERN1","O75460","CHEMBL1163101","Enzyme","0.112041901328"
68	Lysine-specific histone demethylase 1,"KDM1A","O60341","CHEMBL6136","Eraser","0.112041901328"
69	Beta-secretase 1,"BACE1","P56817","CHEMBL4822","Protease","0.112041901328"
70	Endothelin receptor ET-A,"EDNRA","P25101","CHEMBL252","Family A G protein-coupled receptor","0.112041901328"
71	Matrix metalloproteinase 3,"MMP3","P08254","CHEMBL283","Protease","0.112041901328"
72	Atrial natriuretic factor,"NPPA","P01160","CHEMBL1293193","Unclassified protein","0.112041901328"
73	Gamma-secretase,"PSEN2 PSENEN NCSTN APH1A PSEN1 APH1B","P49810 Q9NZ42 Q92542 Q96BI3 P49768 Q8WW43","CHEMBL2094135","Protease","0.112041901328"
74	Serine/threonine-protein kinase PIM1,"PIM1","P11309","CHEMBL2147","Kinase","0.112041901328"
75	Serine/threonine-protein kinase PIM2,"PIM2","Q9P1W9","CHEMBL4523","Kinase","0.112041901328"
76	Protein arginine N-methyltransferase 5,"PRMT5","O14744","CHEMBL1795116","Writer","0.112041901328"
77	Histone-lysine N-methyltransferase, H3 lysine-79 specific,"DOT1L","Q8TEK3","CHEMBL1795117","Writer","0.112041901328"
78	DNA (cytosine-5)-methyltransferase 1,"DNMT1","P26358","CHEMBL1993","Writer","0.112041901328"
79	Nicotinamide N-methyltransferase,"NNMT","P40261","CHEMBL2346486","Enzyme","0.112041901328"
80	Protein arginine N-methyltransferase 3,"PRMT3","O60678","CHEMBL5891","Writer","0.112041901328"
81	Peptidyl-prolyl cis-trans isomerase NIMA-interacting 1,"PIN1","Q13526","CHEMBL2288","Enzyme","0.112041901328"
82	Aminopeptidase B (by homology),"RNPEP","Q9H4A4","CHEMBL2432","Protease","0.112041901328"
83	Matrix metalloproteinase 2,"MMP2","P08253","CHEMBL333","Protease","0.112041901328"
84	Histone-lysine N-methyltransferase MLL,"KMT2A","Q03164","CHEMBL1293299","Writer","0.112041901328"
85	Histone-lysine N-methyltransferase SUV39H1,"SUV39H1","O43463","CHEMBL1795118","Writer","0.112041901328"
86	Indolethylamine N-methyltransferase,"INMT","O95050","CHEMBL2131","Enzyme","0.112041901328"
87	EZH2/SUZ12/EED/RBBP7/RBBP4,"EZH2","Q15910","CHEMBL2189110","Writer","0.112041901328"
88	Histone-lysine N-methyltransferase EZH1,"EZH1","Q92800","CHEMBL2189116","Writer","0.112041901328"
89	Histone-lysine N-methyltransferase SETDB1,"SETDB1","Q15047","CHEMBL2321646","Writer","0.112041901328"
90	Histone-arginine methyltransferase CARM1,"CARM1","Q86X55","CHEMBL5406","Writer","0.112041901328"
91	Protein-arginine N-methyltransferase 1,"PRMT1","Q99873","CHEMBL5524","Writer","0.112041901328"
92	Histone-lysine N-methyltransferase, H3 lysine-9 specific 5,"EHMT1","Q9H9B1","CHEMBL6031","Writer","0.112041901328"
93	Histone-lysine N-methyltransferase, H3 lysine-9 specific 3,"EHMT2","Q96KQ7","CHEMBL6032","Writer","0.112041901328"
94	DNA (cytosine-5)-methyltransferase 3B,"DNMT3B","Q9UBC3","CHEMBL6095","Reader","0.112041901328"
95	Protein-tyrosine phosphatase 1C,"PTPN6","P29350","CHEMBL3166","Phosphatase","0.112041901328"

96	N-acylsphingosine-amidohydrolase (by homology),"NAAA","Q02083","CHEMBL4349","Enzyme","0.112041901328"
97	Sigma opioid receptor,"SIGMAR1","Q99720","CHEMBL287","Membrane receptor","0.112041901328"
98	Thrombin,"F2","P00734","CHEMBL204","Protease","0.112041901328"
99	Metabotropic glutamate receptor 2 (by homology),"GRM2","Q14416","CHEMBL5137","Family C G protein-coupled receptor","0.112041901328"
100	Cathepsin S,"CTSS","P25774","CHEMBL2954","Protease","0.112041901328"

Table S7. Predicted targets for QF peptide. Red font indicates 15 most likely targets.

Order:	"Target","Common name","Uniprot ID","ChEMBL ID","Target Class","Probability"
1	Angiotensin-converting enzyme,"ACE","P12821","CHEMBL1808","Protease","0.237885167579"
2	Calpain 1,"CAPN1","P07384","CHEMBL3891","Protease","0.229685699326"
3	Tyrosyl-tRNA synthetase,"YARS","P54577","CHEMBL3179","Enzyme","0.139061946948"
4	Cyclooxygenase-2,"PTGS2","P35354","CHEMBL230","Oxidoreductase","0.114337558605"
5	Neprilysin,"MME","P08473","CHEMBL1944","Protease","0.114337558605"
6	Delta opioid receptor,"OPRD1","P41143","CHEMBL236","Family A G protein-coupled receptor","0.114337558605"
7	Integrin alpha-V/beta-3,"ITGAV ITGB3","P06756 P05106","CHEMBL1907598","Membrane receptor","0.106099949133"
8	Leucine aminopeptidase,"LAP3","P28838","CHEMBL3965","Protease","0.106099949133"
9	Aminopeptidase N,"ANPEP","P15144","CHEMBL1907","Protease","0.106099949133"
10	Integrin alpha-4/beta-1,"ITGB1 ITGA4","P05556 P13612","CHEMBL1907599","Membrane receptor","0.106099949133"
11	Inhibitor of apoptosis protein 3,"XIAP","P98170","CHEMBL4198","Other cytosolic protein","0.106099949133"
12	HLA class I histocompatibility antigen A-3,"HLA-A","P04439","CHEMBL2632","Surface antigen","0.106099949133"
13	Protein farnesyltransferase,"FNTA FNTB","P49354 P49356","CHEMBL2094108","Enzyme","0.106099949133"
14	Mu opioid receptor (by homology),"OPRM1","P35372","CHEMBL233","Family A G protein-coupled receptor","0.106099949133"
15	Solute carrier family 22 member 6 (by homology),"SLC22A6","Q4U2R8","CHEMBL1641347","Electrochemical transporter","0.106099949133"
16	Oligopeptide transporter small intestine isoform,"SLC15A1","P46059","CHEMBL4605","Electrochemical transporter","0.0978745343258"
17	Neurokinin 1 receptor (by homology),"TACR1","P25103","CHEMBL249","Family A G protein-coupled receptor","0.0978745343258"
18	Nitric oxide synthase, inducible,"NOS2","P35228","CHEMBL4481","Enzyme","0.0978745343258"
19	Cyclin-dependent kinase 4/cyclin D1,"CCND1 CDK4","P24385 P11802","CHEMBL1907601","Kinase","0.0978745343258"
20	CDK2/Cyclin A,"CCNA2 CDK2","P20248 P24941","CHEMBL3038469","Kinase","0.0978745343258"
21	Neurotensin receptor 1 (by homology),"NTSR1","P30989","CHEMBL4123","Family A G protein-coupled receptor","0.0978745343258"
22	Leukotriene A4 hydrolase,"LTA4H","P09960","CHEMBL4618","Protease","0.0978745343258"
23	Tyrosine-protein kinase SRC,"SRC","P12931","CHEMBL267","Kinase","0.0978745343258"
24	Matrix metalloproteinase 2,"MMP2","P08253","CHEMBL333","Protease","0.0978745343258"
25	HMG-CoA reductase,"HMGCR","P04035","CHEMBL402","Oxidoreductase","0.0978745343258"
26	Aminopeptidase B (by homology),"RNPEP","Q9H4A4","CHEMBL2432","Protease","0.0978745343258"
27	Beta-secretase 1,"BACE1","P56817","CHEMBL4822","Protease","0.0978745343258"
28	Formyl peptide receptor 1,"FPR1","P21462","CHEMBL3359","Family A G protein-coupled receptor","0.0978745343258"
29	Integrin alpha-IIb/beta-3,"ITGA2B ITGB3","P08514 P05106","CHEMBL2093869","Membrane receptor","0.0978745343258"
30	Chromobox protein homolog 7,"CBX7","O95931","CHEMBL1764946","Reader","0.0978745343258","0 / 19Â Â Â Â Â "

31	E3 SUMO-protein ligase CBX4,"CBX4","O00257","CHEMBL3232685","Enzyme","0.0978745343258"
32	Neuropeptide FF receptor 1,"NPFFR1","Q9GZQ6","CHEMBL5951","Family A G protein-coupled receptor","0.0978745343258"
33	Neuropeptide FF receptor 2,"NPFFR2","Q9Y5X5","CHEMBL5952","Family A G protein-coupled receptor","0.0978745343258"
34	Dipeptidyl peptidase IV,"DPP4","P27487","CHEMBL284","Protease","0.0978745343258"
35	Neurotensin receptor 2,"NTSR2","O95665","CHEMBL2514","Family A G protein-coupled receptor","0.0978745343258"
36	Dopamine transporter (by homology),"SLC6A3","Q01959","CHEMBL238","Electrochemical transporter","0.0978745343258"
37	Calcitonin gene-related peptide type 1 receptor,"CALCRL","Q16602","CHEMBL3798","Family B G protein-coupled receptor","0.0978745343258"
38	Kappa Opioid receptor,"OPRK1","P41145","CHEMBL237","Family A G protein-coupled receptor","0.0978745343258"
39	Signal transducer and activator of transcription 3,"STAT3","P40763","CHEMBL4026","Transcription factor","0.0978745343258"
40	Proenkephalin B,"PDYN","P01213","CHEMBL2227","Other ion channel","0.0978745343258"
41	Epoxide hydratase,"EPHX2","P34913","CHEMBL2409","Protease","0.0978745343258"
42	Beta secretase 2,"BACE2","Q9Y5Z0","CHEMBL2525","Protease","0.0978745343258"
43	Thyrotropin-releasing hormone receptor (by homology),"TRHR","P34981","CHEMBL1810","Family A G protein-coupled receptor","0.0978745343258"
44	Bradykinin B1 receptor,"BDKRB1","P46663","CHEMBL4308","Family A G protein-coupled receptor","0.0978745343258"
45	Ribonucleoside-diphosphate reductase M1 chain (by homology),"RRM1","P23921","CHEMBL1830","Oxidoreductase","0.0978745343258"
46	Renin,"REN","P00797","CHEMBL286","Protease","0.0978745343258"
47	Dipeptidyl peptidase VIII,"DPP8","Q6V1X1","CHEMBL4657","Protease","0.0978745343258"
48	Neurokinin 2 receptor,"TACR2","P21452","CHEMBL2327","Family A G protein-coupled receptor","0.0978745343258"
49	Carboxypeptidase B2 isoform A,"CPB2","Q96IY4","CHEMBL3419","Protease","0.0978745343258"
50	NAD-dependent deacetylase sirtuin 2,"SIRT2","Q8IXJ6","CHEMBL4462","Eraser","0.0978745343258"
51	Kallikrein 1,"KLK1","P06870","CHEMBL2319","Protease","0.0978745343258"
52	Integrin alpha-5/beta-1,"ITGB1 ITGA5","P05556 P08648","CHEMBL2095226","Membrane receptor","0.0978745343258"
53	Baculoviral IAP repeat-containing protein 3,"BIRC3","Q13489","CHEMBL5335","Enzyme","0.0978745343258"
54	Baculoviral IAP repeat-containing protein 2,"BIRC2","Q13490","CHEMBL5462","Enzyme","0.0978745343258"
55	Vasopressin V1a receptor (by homology),"AVPR1A","P37288","CHEMBL1889","Family A G protein-coupled receptor","0.0978745343258"
56	Oxytocin receptor (by homology),"OXTR","P30559","CHEMBL2049","Family A G protein-coupled receptor","0.0978745343258"
57	MAP kinase-activated protein kinase 2,"MAPKAPK2","P49137","CHEMBL2208","Kinase","0.0978745343258"
58	Xaa-Pro dipeptidase,"PEPD","P12955","CHEMBL4185","Protease","0.0978745343258"
59	Xaa-Pro aminopeptidase 2,"XPNPEP2","O43895","CHEMBL4610","Protease","0.0978745343258"
60	Angiotensin-converting enzyme 2,"ACE2","Q9BYF1","CHEMBL3736","Protease","0.0978745343258"
61	Disks large homolog 4,"DLG4","P78352","CHEMBL5666","Unclassified protein","0.0978745343258"
62	Sigma opioid receptor,"SIGMAR1","Q99720","CHEMBL287","Membrane receptor","0.0978745343258"
63	Thrombin,"F2","P00734","CHEMBL204","Protease","0.0978745343258"

64	Dipeptidyl peptidase IX,"DPP9","Q86TI2","CHEMBL4793","Protease","0.0978745343258"
65	Endothelin receptor ET-A,"EDNRA","P25101","CHEMBL252","Family A G protein-coupled receptor","0.0978745343258"
66	Pyroglutamylated RFamide peptide receptor,"QRFPR","Q96P65","CHEMBL5852","Family A G protein-coupled receptor","0.0978745343258"
67	Sphingosine kinase 1,"SPHK1","Q9NYA1","CHEMBL4394","Enzyme","0.0978745343258"
68	Atrial natriuretic factor,"NPPA","P01160","CHEMBL1293193","Unclassified protein","0.0978745343258"
69	HLA class II histocompatibility antigen DRB1-1,"HLA-DRB1","P04229","CHEMBL1943","Surface antigen","0.0978745343258"
70	Galanin receptor 1 (by homology),"GALR1","P47211","CHEMBL4894","Family A G protein-coupled receptor","0.0978745343258"
71	Neurotensin receptor 3,"SORT1","Q99523","CHEMBL3091","Membrane receptor","0.0978745343258"
72	Endothelin-converting enzyme 1,"ECE1","P42892","CHEMBL4791","Protease","0.0978745343258"
73	Gamma-secretase,"PSEN2 PSENEN NCSTN APH1A PSEN1 APH1B","P49810 Q9NZ42 Q92542 Q96BI3 P49768 Q8WW43","CHEMBL2094135","Protease","0.0978745343258"
74	Cholecystokinin B receptor,"CCKBR","P32239","CHEMBL298","Family A G protein-coupled receptor","0.0978745343258"
75	Xaa-Pro aminopeptidase 1,"XPNPEP1","Q9NQW7","CHEMBL3782","Protease","0.0978745343258"
76	C-X-C chemokine receptor type 7,"ACKR3","P25106","CHEMBL2010631","Family A G protein-coupled receptor","0.0978745343258"
77	Max-like protein X,"MLX","Q9UH92","CHEMBL2062357","Unclassified protein","0.0978745343258"
78	Furin,"FURIN","P09958","CHEMBL2611","Protease","0.0978745343258"
79	Cathepsin D,"CTSD","P07339","CHEMBL2581","Protease","0.0978745343258"
80	Cathepsin E,"CTSE","P14091","CHEMBL3092","Protease","0.0978745343258"
81	Vasopressin V2 receptor (by homology),"AVPR2","P30518","CHEMBL1790","Family A G protein-coupled receptor","0.0978745343258"
82	Lysine-specific demethylase 4A,"KDM4A","O75164","CHEMBL5896","Eraser","0.0978745343258"
83	Lysine-specific demethylase 4C,"KDM4C","Q9H3R0","CHEMBL6175","Eraser","0.0978745343258"
84	Alkaline ceramidase 2,"ACER2","Q5QJU3","CHEMBL2331067","Enzyme","0.0978745343258"
85	Acid ceramidase,"ASAH1","Q13510","CHEMBL5463","Enzyme","0.0978745343258"
86	Cyclin-dependent kinase 2,"CDK2","P24941","CHEMBL301","Kinase","0.0978745343258"
87	Ghrelin receptor,"GHSR","Q92847","CHEMBL4616","Family A G protein-coupled receptor","0.0978745343258"
88	MAP kinase ERK2,"MAPK1","P28482","CHEMBL4040","Kinase","0.0978745343258"
89	Peptidyl-glycine alpha-amidating monooxygenase,"PAM","P19021","CHEMBL2544","Enzyme","0.0978745343258"
90	Mas-related G-protein coupled receptor member X1 (by homology),"MRGPRX1","Q96LB2","CHEMBL5850","Unclassified protein","0.0978745343258"
91	Ephrin type-A receptor 2,"EPHA2","P29317","CHEMBL2068","Kinase","0.0978745343258"
92	Hydroxycarboxylic acid receptor 2,"HCAR2","Q8TDS4","CHEMBL3785","Family A G protein-coupled receptor","0.0978745343258"
93	Appetite-regulating hormone,"GHRL","Q9UBU3","CHEMBL1921664","Unclassified protein","0.0978745343258"
94	HLA class II histocompatibility antigen DRB3-1,"HLA-DRB3","P79483","CHEMBL3460","Surface antigen","0.0978745343258"
95	Serine/threonine-protein kinase MST2,"STK3","Q13188","CHEMBL4708","Kinase","0.0978745343258"

96	Serine/threonine-protein kinase Aurora-A,"AURKA","O14965","CHEMBL4722","Kinase","0.0978745343258"
97	Melanocortin receptor 4,"MC4R","P32245","CHEMBL259","Family A G protein-coupled receptor","0.0978745343258"
98	Melanocortin receptor 3,"MC3R","P41968","CHEMBL4644","Family A G protein-coupled receptor","0.0978745343258"
99	Cyclophilin A,"PPIA","P62937","CHEMBL1949","Isomerase","0.0978745343258"
100	Serine/threonine-protein kinase PIM1,"PIM1","P11309","CHEMBL2147","Kinase","0.0978745343258"

Table S8. Predicted targets for DF peptide. Red font indicates 15 most likely targets.

Order:	"Target","Common name","Uniprot ID","ChEMBL ID","Target Class","Probability"
1	Calpain 1,"CAPN1","P07384","CHEMBL3891","Protease","0.149732593856"
2	Angiotensin-converting enzyme,"ACE","P12821","CHEMBL1808","Protease","0.116965063224"
3	Neprilysin,"MME","P08473","CHEMBL1944","Protease","0.108770969359"
4	Ribonucleoside-diphosphate reductase M1 chain (by homology),"RRM1","P23921","CHEMBL1830","Oxidoreductase","0.100578902067"
5	Neurokinin 2 receptor,"TACR2","P21452","CHEMBL2327","Family A G protein-coupled receptor","0.100578902067"
6	Beta-secretase 1,"BACE1","P56817","CHEMBL4822","Protease","0.100578902067"
7	Delta opioid receptor (by homology),"OPRD1","P41143","CHEMBL236","Family A G protein-coupled receptor","0.100578902067"
8	Tyrosyl-tRNA synthetase,"YARS","P54577","CHEMBL3179","Enzyme","0.100578902067"
9	Calcitonin gene-related peptide type 1 receptor,"CALCRL","Q16602","CHEMBL3798","Family B G protein-coupled receptor","0.100578902067"
10	HMG-CoA reductase,"HMGCR","P04035","CHEMBL402","Oxidoreductase","0.100578902067","0 / 14Â Â Â Â Â "
11	Endothelin receptor ET-A,"EDNRA","P25101","CHEMBL252","Family A G protein-coupled receptor","0.100578902067"
12	Inhibitor of apoptosis protein 3,"XIAP","P98170","CHEMBL4198","Other cytosolic protein","0.100578902067"
13	HLA class I histocompatibility antigen A-3,"HLA-A","P04439","CHEMBL2632","Surface antigen","0.100578902067"
14	Beta secretase 2,"BACE2","Q9Y5Z0","CHEMBL2525","Protease","0.100578902067"
15	Leucine aminopeptidase,"LAP3","P28838","CHEMBL3965","Protease","0.100578902067"
16	Mu opioid receptor (by homology),"OPRM1","P35372","CHEMBL233","Family A G protein-coupled receptor","0.100578902067"
17	Metabotropic glutamate receptor 3,"GRM3","Q14832","CHEMBL2888","Family C G protein-coupled receptor","0.100578902067"
18	Metabotropic glutamate receptor 2,"GRM2","Q14416","CHEMBL5137","Family C G protein-coupled receptor","0.100578902067"
19	Solute carrier family 22 member 6 (by homology),"SLC22A6","Q4U2R8","CHEMBL1641347","Electrochemical transporter","0.100578902067"
20	Gamma-glutamylcysteine synthetase,"GCLC","P48506","CHEMBL4055","Enzyme","0.100578902067"
21	Integrin alpha-4/beta-1,"ITGB1 ITGA4","P05556 P13612","CHEMBL1907599","Membrane receptor","0.100578902067"
22	Kappa Opioid receptor,"OPRK1","P41145","CHEMBL237","Family A G protein-coupled receptor","0.100578902067"
23	Aminopeptidase N,"ANPEP","P15144","CHEMBL1907","Protease","0.100578902067"
24	Glutathione S-transferase kappa 1,"GSTK1","Q9Y2Q3","CHEMBL4491","Enzyme","0.100578902067"
25	Metabotropic glutamate receptor 6,"GRM6","O15303","CHEMBL4573","Family C G protein-coupled receptor","0.100578902067"
26	Proenkephalin B,"PDYN","P01213","CHEMBL2227","Other ion channel","0.100578902067"
27	Peptidyl-prolyl cis-trans isomerase NIMA-interacting 1,"PIN1","Q13526","CHEMBL2288","Enzyme","0.100578902067"
28	Matrix metalloproteinase 3,"MMP3","P08254","CHEMBL283","Protease","0.100578902067"
29	Matrix metalloproteinase 1,"MMP1","P03956","CHEMBL332","Protease","0.100578902067"
30	Formyl peptide receptor 1,"FPR1","P21462","CHEMBL3359","Family A G protein-coupled receptor","0.100578902067"

31	Ephrin type-A receptor 2,"EPHA2","P29317","CHEMBL2068","Kinase","0.100578902067"
32	Tyrosine-protein kinase SRC,"SRC","P12931","CHEMBL267","Kinase","0.100578902067"
33	Chromobox protein homolog 7,"CBX7","O95931","CHEMBL1764946","Reader","0.100578902067"
34	E3 SUMO-protein ligase CBX4,"CBX4","O00257","CHEMBL3232685","Enzyme","0.100578902067"
35	Protein farnesyltransferase,"FNTA FNTB","P49354 P49356","CHEMBL2094108","Enzyme","0.100578902067"
36	Cholecystokinin B receptor,"CCKBR","P32239","CHEMBL298","Family A G protein-coupled receptor","0.100578902067"
37	Angiotensin II receptor,"AGTR2","P50052","CHEMBL4607","Family A G protein-coupled receptor","0.100578902067"
38	Galanin receptor 1 (by homology),"GALR1","P47211","CHEMBL4894","Family A G protein-coupled receptor","0.100578902067"
39	Galanin receptor 2 (by homology),"GALR2","O43603","CHEMBL3176","Family A G protein-coupled receptor","0.100578902067"
40	Neurokinin 1 receptor (by homology),"TACR1","P25103","CHEMBL249","Family A G protein-coupled receptor","0.100578902067"
41	Oligopeptide transporter small intestine isoform,"SLC15A1","P46059","CHEMBL4605","Electrochemical transporter","0.100578902067"
42	Tubulin beta-1 chain,"TUBB1","Q9H4B7","CHEMBL1915","Structural protein","0.100578902067"
43	Epoxide hydratase (by homology),"EPHX2","P34913","CHEMBL2409","Protease","0.100578902067"
44	Glutamate receptor ionotropic, AMPA 2,"GRIA2","P42262","CHEMBL4016","Ligand-gated ion channel","0.100578902067"
45	Excitatory amino acid transporter 2,"SLC1A2","P43004","CHEMBL4973","Electrochemical transporter","0.100578902067"
46	Carboxypeptidase A1,"CPA1","P15085","CHEMBL2088","Protease","0.100578902067"
47	Angiotensin-converting enzyme 2,"ACE2","Q9BYF1","CHEMBL3736","Protease","0.100578902067"
48	HLA class II histocompatibility antigen DRB3-1,"HLA-DRB3","P79483","CHEMBL3460","Surface antigen","0.100578902067"
49	Interleukin-1 beta,"IL1B","P01584","CHEMBL1909490","Secreted protein","0.100578902067"
50	Oxytocin receptor (by homology),"OXTR","P30559","CHEMBL2049","Family A G protein-coupled receptor","0.100578902067"
51	ADAM17,"ADAM17","P78536","CHEMBL3706","Protease","0.100578902067"
52	Glutamate receptor ionotropic kainate 3,"GRIK3","Q13003","CHEMBL3684","Ligand-gated ion channel","0.100578902067"
53	Cyclin-dependent kinase 4/cyclin D1,"CCND1 CDK4","P24385 P11802","CHEMBL1907601","Kinase","0.100578902067"
54	CDK2/Cyclin A,"CCNA2 CDK2","P20248 P24941","CHEMBL3038469","Kinase","0.100578902067"
55	MAP kinase ERK2,"MAPK1","P28482","CHEMBL4040","Kinase","0.100578902067"
56	Neurotensin receptor 1 (by homology),"NTSR1","P30989","CHEMBL4123","Family A G protein-coupled receptor","0.100578902067"
57	Neuropeptide FF receptor 1,"NPFFR1","Q9GZQ6","CHEMBL5951","Family A G protein-coupled receptor","0.100578902067"
58	Neuropeptide FF receptor 2,"NPFFR2","Q9Y5X5","CHEMBL5952","Family A G protein-coupled receptor","0.100578902067"
59	Cathepsin (B and K),"CTSB","P07858","CHEMBL4072","Protease","0.100578902067"
60	HLA class II histocompatibility antigen DRB1-1,"HLA-DRB1","P04229","CHEMBL1943","Surface antigen","0.100578902067"
61	Tryptase beta-1,"TPSAB1","Q15661","CHEMBL2617","Protease","0.100578902067"
62	Sigma opioid receptor,"SIGMAR1","Q99720","CHEMBL287","Membrane receptor","0.100578902067"
63	Atrial natriuretic factor,"NPPA","P01160","CHEMBL1293193","Unclassified protein","0.100578902067"

64	Cyclin-dependent kinase 2/cyclin A,"CDK2 CCNA1 CCNA2","P24941 P78396 P20248","CHEMBL2094128","Other cytosolic protein","0.100578902067"
65	Betaine--homocysteine S-methyltransferase 1,"BHMT","Q93088","CHEMBL4328","Enzyme","0.100578902067"
66	Kallikrein 1,"KLK1","P06870","CHEMBL2319","Protease","0.100578902067"
67	Neurotensin receptor 2,"NTSR2","O95665","CHEMBL2514","Family A G protein-coupled receptor","0.100578902067"
68	Cathepsin L,"CTSL","P07711","CHEMBL3837","Protease","0.100578902067"
69	Cyclooxygenase-2,"PTGS2","P35354","CHEMBL230","Oxidoreductase","0.100578902067"
70	Signal transducer and activator of transcription 3,"STAT3","P40763","CHEMBL4026","Transcription factor","0.100578902067"
71	Integrin alpha-IIb/beta-3,"ITGA2B ITGB3","P08514 P05106","CHEMBL2093869","Membrane receptor","0.100578902067"
72	Histone deacetylase 1,"HDAC1","Q13547","CHEMBL325","Eraser","0.100578902067"
73	Cathepsin K,"CTSK","P43235","CHEMBL268","Protease","0.100578902067"
74	Neurokinin 3 receptor,"TACR3","P29371","CHEMBL4429","Family A G protein-coupled receptor","0.100578902067"
75	Geranylgeranyl transferase type I,"PGGT1B FNTA","P53609 P49354","CHEMBL2095164","Enzyme","0.100578902067"
76	Elastase 1,"CELA1","Q9UNI1","CHEMBL3000","Protease","0.100578902067"
77	Cathepsin G,"CTSG","P08311","CHEMBL4071","Protease","0.100578902067"
78	Beta-chymotrypsin,"CTRB1","P17538","CHEMBL4796","Protease","0.100578902067"
79	Dipeptidyl peptidase VIII,"DPP8","Q6V1X1","CHEMBL4657","Protease","0.100578902067"
80	Dipeptidyl peptidase I,"CTSC","P53634","CHEMBL2252","Protease","0.100578902067"
81	Bradykinin B1 receptor,"BDKRB1","P46663","CHEMBL4308","Family A G protein-coupled receptor","0.100578902067"

Table S9. Predicted targets for DR peptide. Red font indicates 15 most likely targets.

Order:	"Target","Common name","Uniprot ID","ChEMBL ID","Target Class","Probability"
1	Complement factor B,"CFB","P00751","CHEMBL5731","Protease","0.108770969359"
2	Furin,"FURIN","P09958","CHEMBL2611","Protease","0.108770969359"
3	Integrin alpha-IIb/beta-3,"ITGA2B ITGB3","P08514 P05106","CHEMBL2093869","Membrane receptor","0.108770969359"
4	Integrin alpha-V/beta-5,"ITGB5 ITGAV","P18084 P06756","CHEMBL2096675","Membrane receptor","0.108770969359"
5	Hepatocyte growth factor activator,"HGFAC","Q04756","CHEMBL3351190","Unclassified protein","0.108770969359"
6	Subtilisin/kexin type 6,"PCSK6","P29122","CHEMBL2951","Protease","0.108770969359"
7	Neurotensin receptor 2,"NTSR2","O95665","CHEMBL2514","Family A G protein-coupled receptor","0.108770969359"
8	Neurotensin receptor 1,"NTSR1","P30989","CHEMBL4123","Family A G protein-coupled receptor","0.108770969359"
9	Disintegrin and metalloproteinase domain-containing protein 8,"ADAM8","P78325","CHEMBL5665","Protease","0.100578902067"
10	Neuropilin-1 (by homology),"NRP1","O14786","CHEMBL5174","Secreted protein","0.100578902067"
11	Thrombin and coagulation factor X,"F10","P00742","CHEMBL244","Protease","0.100578902067"
12	WD repeat-containing protein 5,"WDR5","P61964","CHEMBL1075317","Unclassified protein","0.100578902067"
13	c-Jun N-terminal kinase 1,"MAPK8","P45983","CHEMBL2276","Kinase","0.100578902067"
14	Epoxide hydratase (by homology),"EPHX2","P34913","CHEMBL2409","Protease","0.100578902067"
15	Integrin alpha-V/beta-3,"ITGAV ITGB3","P06756 P05106","CHEMBL1907598","Membrane receptor","0.100578902067"
16	Integrin alpha-5/beta-1,"ITGB1 ITGA5","P05556 P08648","CHEMBL2095226","Membrane receptor","0.100578902067"
17	Renin,"REN","P00797","CHEMBL286","Protease","0.100578902067"
18	Transcription factor AP1,"FOS JUN","P01100 P05412","CHEMBL2111421","Transcription factor","0.100578902067"
19	Plasminogen,"PLG","P00747","CHEMBL1801","Protease","0.100578902067"
20	Thrombin,"F2","P00734","CHEMBL204","Protease","0.100578902067"
21	Proteinase-activated receptor 2,"F2RL1","P55085","CHEMBL5963","Family A G protein-coupled receptor","0.100578902067"
22	Caspase-8,"CASP8","Q14790","CHEMBL3776","Protease","0.100578902067"
23	HLA class I histocompatibility antigen A-3,"HLA-A","P04439","CHEMBL2632","Surface antigen","0.100578902067"
24	Vitronectin receptor alpha,"ITGAV","P06756","CHEMBL3660","Membrane receptor","0.100578902067"
25	Disks large homolog 4,"DLG4","P78352","CHEMBL5666","Unclassified protein","0.100578902067"
26	Sodium/glucose cotransporter 1,"SLC5A1","P13866","CHEMBL4979","Electrochemical transporter","0.100578902067"
27	Cathepsin K,"CTSK","P43235","CHEMBL268","Protease","0.100578902067"
28	Transmembrane protease serine 6,"TMPRSS6","Q8IU80","CHEMBL1795139","Protease","0.100578902067"
29	Serine protease hepsin,"HPN","P05981","CHEMBL2079849","Protease","0.100578902067"
30	Cathepsin (H and K),"CTSH","P09668","CHEMBL2225","Protease","0.100578902067"

31	Matriptase,"ST14","Q9Y5Y6","CHEMBL3018","Protease","0.100578902067"
32	Cathepsin L,"CTSL","P07711","CHEMBL3837","Protease","0.100578902067"
33	Calpain 1,"CAPN1","P07384","CHEMBL3891","Protease","0.100578902067"
34	Cathepsin (B and K),"CTSB","P07858","CHEMBL4072","Protease","0.100578902067"
35	Cathepsin D,"CTSD","P07339","CHEMBL2581","Protease","0.100578902067"
36	Beta-secretase 1,"BACE1","P56817","CHEMBL4822","Protease","0.100578902067"
37	Pepsinogen C (by homology),"PGC","P20142","CHEMBL2136","Protease","0.100578902067"
38	Cathepsin E,"CTSE","P14091","CHEMBL3092","Protease","0.100578902067"
39	Pepsin A,"PGA5","P0DJD9","CHEMBL3295","Protease","0.100578902067"
40	ADAM9,"ADAM9","Q13443","CHEMBL5982","Protease","0.100578902067"
41	Inhibitor of apoptosis protein 3,"XIAP","P98170","CHEMBL4198","Other cytosolic protein","0.100578902067"
42	Dipeptidyl peptidase IV,"DPP4","P27487","CHEMBL284","Protease","0.100578902067"

Table S10. Predicted targets for GR peptide. Red font indicates 15 most likely targets.

Order:	"Target","Common name","Uniprot ID","ChEMBL ID","Target Class","Probability"
1	Complement factor B,"CFB","P00751","CHEMBL5731","Protease","0.112450964818"
2	Furin,"FURIN","P09958","CHEMBL2611","Protease","0.103761755413"
3	Neurotensin receptor 2,"NTSR2","O95665","CHEMBL2514","Family A G protein-coupled receptor","0.0864426933852"
4	Neurotensin receptor 1,"NTSR1","P30989","CHEMBL4123","Family A G protein-coupled receptor","0.0864426933852"
5	Subtilisin/kexin type 6,"PCSK6","P29122","CHEMBL2951","Protease","0.0864426933852"
6	Hepatocyte growth factor activator,"HGFAC","Q04756","CHEMBL3351190","Unclassified protein","0.0864426933852"
7	Neuropilin-1 (by homology),"NRP1","O14786","CHEMBL5174","Secreted protein","0.0690974435253"
8	WD repeat-containing protein 5,"WDR5","P61964","CHEMBL1075317","Unclassified protein","0.0690974435253"
9	Epoxide hydratase (by homology),"EPHX2","P34913","CHEMBL2409","Protease","0.0690974435253"
10	Nitric oxide synthase, inducible,"NOS2","P35228","CHEMBL4481","Enzyme","0.0604245879294"
11	Nitric-oxide synthase, brain,"NOS1","P29475","CHEMBL3568","Enzyme","0.0604245879294"
12	Proteinase-activated receptor 2,"F2RL1","P55085","CHEMBL5963","Family A G protein-coupled receptor","0.0604245879294"
13	Transmembrane protease serine 6,"TMPRSS6","Q8IU80","CHEMBL1795139","Protease","0.0604245879294"
14	Plasminogen,"PLG","P00747","CHEMBL1801","Protease","0.0604245879294"
15	Serine protease hepsin,"HPN","P05981","CHEMBL2079849","Protease","0.0604245879294"
16	Cathepsin (H and K),"CTSH","P09668","CHEMBL2225","Protease","0.0604245879294"
17	Matriptase,"ST14","Q9Y5Y6","CHEMBL3018","Protease","0.0604245879294"
18	Cathepsin L,"CTSL","P07711","CHEMBL3837","Protease","0.0604245879294"
19	Calpain 1,"CAPN1","P07384","CHEMBL3891","Protease","0.0604245879294"
20	Cathepsin (B and K),"CTSB","P07858","CHEMBL4072","Protease","0.0604245879294"
21	Thrombin,"F2","P00734","CHEMBL204","Protease","0.0604245879294"
22	Nitric-oxide synthase, endothelial,"NOS3","P29474","CHEMBL4803","Enzyme","0.0604245879294"
23	Dipeptidyl peptidase II,"DPP7","Q9UHL4","CHEMBL3976","Protease","0.0604245879294"
24	Dipeptidyl peptidase VIII,"DPP8","Q6V1X1","CHEMBL4657","Protease","0.0604245879294"
25	Dipeptidyl peptidase IV,"DPP4","P27487","CHEMBL284","Protease","0.0604245879294"
26	Integrin alpha-5/beta-1,"ITGB1 ITGA5","P05556 P08648","CHEMBL2095226","Membrane receptor","0.0604245879294"
27	c-Jun N-terminal kinase 1,"MAPK8","P45983","CHEMBL2276","Kinase","0.0604245879294"
28	Integrin alpha-V/beta-3,"ITGAV ITGB3","P06756 P05106","CHEMBL1907598","Membrane receptor","0.0604245879294"
29	C3a anaphylatoxin chemotactic receptor,"C3AR1","Q16581","CHEMBL4761","Family A G protein-coupled receptor","0.0604245879294"
30	Integrin alpha-V/beta-5,"ITGB5 ITGAV","P18084 P06756","CHEMBL2096675","Membrane receptor","0.0604245879294"

31	Muscarinic acetylcholine receptor M4,"CHRM4","P08173","CHEMBL1821","Family A G protein-coupled receptor","0.0604245879294"
32	Muscarinic acetylcholine receptor M2,"CHRM2","P08172","CHEMBL211","Family A G protein-coupled receptor","0.0604245879294"
33	Muscarinic acetylcholine receptor M1,"CHRM1","P11229","CHEMBL216","Family A G protein-coupled receptor","0.0604245879294"
34	Tripeptidyl-peptidase 2,"TPP2","P29144","CHEMBL6156","Protease","0.0604245879294"
35	Disintegrin and metalloproteinase domain-containing protein 8,"ADAM8","P78325","CHEMBL5665","Protease","0.0604245879294"
36	HLA class I histocompatibility antigen A-3,"HLA-A","P04439","CHEMBL2632","Surface antigen","0.0604245879294"
37	Inhibitor of apoptosis protein 3,"XIAP","P98170","CHEMBL4198","Other cytosolic protein","0.0604245879294"
38	Thrombin and coagulation factor X,"F10","P00742","CHEMBL244","Protease","0.0604245879294"
39	Tryptase beta-1,"TPSAB1","Q15661","CHEMBL2617","Protease","0.0604245879294"
